# RE-MIND2: comparative effectiveness of tafasitamab plus lenalidomide versus polatuzumab vedotin/bendamustine/rituximab (pola-BR), CAR-T therapies, and lenalidomide/rituximab (R2) based on real-world data in patients with relapsed/refractory diffuse large B-cell lymphoma

**DOI:** 10.1007/s00277-023-05196-4

**Published:** 2023-05-12

**Authors:** Grzegorz S. Nowakowski, Dok Hyun Yoon, Patrizia Mondello, Erel Joffe, Anthea Peters, Isabelle Fleury, Richard Greil, Matthew Ku, Reinhard Marks, Kibum Kim, Pier Luigi Zinzani, Judith Trotman, Lorenzo Sabatelli, Eva E. Waltl, Mark Winderlich, Andrea Sporchia, Nuwan C. Kurukulasuriya, Raul Cordoba, Georg Hess, Gilles Salles

**Affiliations:** 1 Division of Hematology, Mayo Clinic, Rochester, MN USA; 2 Department of Oncology, Asan Medical Center, Songpa-gu, Seoul, South Korea; 3 Department of Medicine, Memorial Sloan Kettering Cancer Center, New York, NY USA; 4 Department of Oncology, University of Alberta, Edmonton, Alberta Canada; 5 Maisonneuve-Rosemont Hospital, Institute of Hematology, Oncology and Cell Therapy, Montreal University, Montreal, Canada; 6 Paracelsus Medical University Salzburg, Salzburg Cancer Research Institute-CCCIT, and Cancer Cluster Salzburg, Salzburg, Austria; 7 Department of Haematology, St Vincent’s Hospital and University of Melbourne, Melbourne, Victoria Australia; 8 University Hospital Freiburg Internal Medicine I, Freiburg im Breisgau, Germany; 9 University of Utah, Salt Lake City, UT USA; 10 University of Illinois at Chicago, Chicago, IL USA; 11 IRCCS Azienda Ospedaliero-Universitaria di Bologna, Istituto di Ematologia “Seràgnoli”, Bologna, Italy; 12 Dipartimento di Medicina Specialistica, Diagnostica e Sperimentale Università di Bologna, Bologna, Italy; 13 Haematology Department, Concord Repatriation General Hospital, University of Sydney, Concord, NSW Australia; 14 Incyte Biosciences International Sàrl, Morges, Switzerland; 15 MorphoSys AG, Planegg, Germany; 16 MorphoSys AG, Boston, MA USA; 17 Department of Hematology, Fundacion Jimenez Diaz University Hospital, Health Research Institute IISFJD, Madrid, Spain; 18 Department of Hematology, Oncology and Pneumology, University Medical School of the Johannes Gutenberg-University Mainz, Mainz, Germany

**Keywords:** Tafasitamab, Lenalidomide, DLBCL, Real-world, Relapsed/Refractory

## Abstract

**Abstract:**

RE-MIND2 (NCT04697160) compared patient outcomes from the L-MIND (NCT02399085) trial of tafasitamab+lenalidomide with those of patients treated with other therapies for relapsed/refractory (R/R) diffuse large B-cell lymphoma (DLBCL) who are autologous stem cell transplant ineligible. We present outcomes data for three pre-specified treatments not assessed in the primary analysis.

Data were retrospectively collected from sites in North America, Europe, and the Asia Pacific region. Patients were aged ≥18 years with histologically confirmed DLBCL and received ≥2 systemic therapies for DLBCL (including ≥1 anti-CD20 therapy). Patients enrolled in the observational and L-MIND cohorts were matched using propensity score-based 1:1 nearest-neighbor matching, balanced for six covariates. Tafasitamab+lenalidomide was compared with polatuzumab vedotin+bendamustine+rituximab (pola-BR), rituximab+lenalidomide (R2), and CD19-chimeric antigen receptor T-cell (CAR-T) therapies. The primary endpoint was overall survival (OS). Secondary endpoints included treatment response and progression-free survival.

From 200 sites, 3,454 patients were enrolled in the observational cohort. Strictly matched patient pairs consisted of tafasitamab+lenalidomide versus pola-BR (*n* = 24 pairs), versus R2 (*n* = 33 pairs), and versus CAR-T therapies (*n* = 37 pairs). A significant OS benefit was observed with tafasitamab+lenalidomide versus pola-BR (HR: 0.441; *p* = 0.034) and R2 (HR: 0.435; *p =* 0.012). Comparable OS was observed in tafasitamab+lenalidomide and CAR-T cohorts (HR: 0.953, *p* = 0.892).

Tafasitamab+lenalidomide appeared to improve survival outcomes versus pola-BR and R2, and comparable outcomes were observed versus CAR-T. Although based on limited patient numbers, these data may help to contextualize emerging therapies for R/R DLBCL.

**Clinical trial registration:**

NCT04697160 (January 6, 2021)

**Supplementary Information:**

The online version contains supplementary material available at 10.1007/s00277-023-05196-4.

## Introduction

The Fc-modified humanized anti-CD19 monoclonal antibody tafasitamab, combined with the immunomodulatory drug lenalidomide, was demonstrated to be effective and well-tolerated for autologous stem cell transplant (ASCT)-ineligible patients with relapsed/refractory (R/R) diffuse large B-cell lymphoma (DLBCL) in the single-arm phase II L-MIND study (NCT02399085) [1]. After 3 years of follow-up, the objective response rate (ORR) was 57.5%, with a complete response (CR) occurring in 40% of patients; responses were durable, with a median duration of response (DoR) of 44 months and a median overall survival (OS) of 33.5 months [2]. The combination was granted accelerated approval by the United States (US) Food and Drug Administration (FDA) in July 2020 for adult patients with R/R DLBCL, ineligible for ASCT [3]. Conditional marketing authorization, in the same setting, was granted in August 2021 by the European Medicines Agency and Health Canada [4, 5]. Tafasitamab plus lenalidomide is included in the NCCN Clinical Practice Guidelines in Oncology (NCCN Guidelines) from National Comprehensive Cancer Network (NCCN) as a preferred second-line therapy option for ASCT-ineligible adult patients with R/R DLBCL [6]. There is no universal standard of care for this patient population, which includes other chemo-immunotherapy regimens such as bendamustine plus rituximab (BR), rituximab plus gemcitabine and oxaliplatin (R-GemOx), and rituximab plus lenalidomide (R2) [6–9]. In addition to tafasitamab plus lenalidomide, recent additions to the therapeutic armamentarium for R/R DLBCL include the antibody–drug conjugate (ADC) polatuzumab vedotin (an anti-CD79b ADC combined with BR [pola-BR]) [10], indicated as a preferred regimen option in NCCN Guidelines [6]. The single-agent drug Selinexor [11], the ADC loncastuximab tesirine [12], and the chimeric antigen receptor T-cell (CAR-T) therapies lisocabtagene maraleucel (FDA approved only), tisagenlecleucel, and axicabtagene ciloleucel [13–16], have been approved as third-line treatments [6].

Assessing efficacy and safety of novel therapies in the R/R DLBCL setting in a range of head-to-head randomized trials would be time-consuming and costly, and may ultimately delay patients’ access to novel treatments with improved clinical benefits [17, 18]. Addressing this scenario, regulators have produced guidance for utilizing real-world data (RWD) to supplement clinical trial data as part of the regulatory approval process [19–21]. Moreover, RWD have the potential to provide context for treatments assessed in single-arm clinical trials by serving as a control, thus facilitating indirect assessments of comparative effectiveness [22]. RE-MIND2 (NCT04697160) is a prospectively designed comparative effectiveness study assessing the efficacy of tafasitamab plus lenalidomide using a retrospective, observational, matched-cohort approach. The control arms included patients who received treatment for R/R DLBCL in the real-world setting. By generating historical controls for the tafasitamab plus lenalidomide combination, assessed prospectively in the single-arm L-MIND trial (NCT02399085), RE-MIND2 allows a contextualization of patient outcomes from a non-randomized study that may support healthcare professionals’ therapeutic decision-making. The primary analysis of RE-MIND2 presented comparative outcomes data for closely matched cohorts receiving tafasitamab plus lenalidomide with patient cohorts who received BR, R-GemOx, and a cohort of pooled systemic therapies administered for R/R DLBCL. Tafasitamab plus lenalidomide was associated with significantly improved OS and progression-free survival (PFS), as well as DoR, compared with the comparator therapy cohorts [23].

To compare the effectiveness of tafasitamab plus lenalidomide and of more recently approved NCCN/European Society for Medical Oncology (ESMO)-listed treatments for R/R DLBCL, the study methodology was expanded to account for the lower number of patients enrolled in RE-MIND2 who received these therapies. Here, we present an expanded analysis to follow the primary RE-MIND2 analysis, presenting comparative outcomes of tafasitamab plus lenalidomide versus pola-BR, R2, and CAR-T therapies.

## Methods

### Data collection and patients

Study sites (academic hospitals, public hospitals, and private practices) from 12 countries across Europe, North America, and the Asia Pacific region were selected based on the completeness of data and the number of patients with R/R DLBCL in an institution’s health records. Data were collected using electronic health records, with review of hard-copy patient charts performed as needed, from patients diagnosed with DLBCL between 2010 and 2020.

Eligibility criteria for the observational cohort were based on key eligibility criteria from the L-MIND trial [1]. Accordingly, patients enrolled in the observational cohort had histologically confirmed DLBCL, were aged ≥18 years at initial diagnosis and had received at least two systemic anti-DLBCL regimens, including at least one anti-CD20 containing therapy. Non-eligibility criteria for the observational cohort were central nervous system (CNS) involvement at initial DLBCL diagnosis, prior allogenic transplant, prior treatment with a CD19-targeted therapy or immunomodulatory drugs (e.g., thalidomide, lenalidomide) as frontline DLBCL therapy, having previously received an allogenic stem cell transplant, a history of malignancies other than DLBCL (unless disease free ≥5 years prior to inclusion), having previously received tafasitamab as any line of therapy, and human immunodeficiency virus positive status (sites in Taiwan only).

### Study design

Data were collected from health records at each study site and examined to identify eligible patients. Eligible patients were then included in the study database and patient-level matching performed between the L-MIND population and real-world patients, who received the pre-specified treatments of interest. Comparative populations were balanced using prognostically relevant baseline characteristics. The observation timeframe for patients in L-MIND was from March 2016 to November 2017, and for patients who received pola-BR, R2, and CAR-T therapies from December 2015 to July 2020, December 2013 to April 2020, and April 2016 to September 2020, respectively.

Data collected from patient health records consisted of: date and histological subtype of initial DLBCL diagnosis, demographics, information for baseline covariates (see following), history of cancers other than DLBCL, DLBCL therapies administered and their efficacy outcomes, treatment details (i.e., start date, stop date, or discontinuation and reason [e.g., adverse event]), reasons for ASCT ineligibility, response assessment criteria used (e.g., Cheson 1999, 2007, 2014 [24–26]), Eastern Cooperative Oncology Group performance status (ECOG PS) (when available), patient survival information, bone marrow involvement, and information on tumor biopsies. After data collection, efficacy outcomes were compared in matched populations consisting of patients who received tafasitamab plus lenalidomide (in L-MIND), and patients from observational cohorts treated with pola-BR, R2, or CAR-T therapies.

In the observational cohorts, an index date was assigned for each patient according to therapy line, based on the first record of a systemically administered therapy for R/R DLBCL (start of second-, third-, or fourth-line treatment). Further details assigning therapy lines in the observational cohorts are provided in the Supplementary methods. For patients from L-MIND, the index date was the date of the first dose of either tafasitamab or lenalidomide. The analysis window for patients in the observational cohort was defined as the time between the index date and end of follow-up (defined as either death or last available medical record for the therapy under consideration, maximum 44 months) (Online Resource Fig. 1). The analysis window for patients from L-MIND was defined as the interval between the index date and the data cut-off date (November 2019; i.e., approximately 2 years after the last patient was enrolled in the study).

### Cohort balancing

The choice of cohort balancing characteristics (covariates) was driven by their clinical relevance and availability in patient records. Six balancing covariates were used relative to nine in the RE-MIND2 primary analysis. The use of six rather than nine covariates was to meet the dual need of including the largest possible number of patient characteristics, while still retaining a meaningful sample of matched patients who had received the treatments of interest. The six cohort balancing covariates were: number of prior therapy lines (1 vs. 2/3) [10]; refractoriness to last therapy (Yes vs. No) [14, 27], described in Supplementary methods; primary refractoriness (Yes vs. No) [14, 27], described in Supplementary methods; prior ASCT (Yes vs. No) [10]; age (as a categorical variable with subgroups <70 vs. ≥70 years) [10, 14, 27]; and ECOG PS (0–1 vs. ≥2) [28]. ECOG PS was not included as a balancing covariate in the previously published primary analysis due to a residual imbalance at baseline [23]; it was instead included as a covariate in a sensitivity analysis. The additional balancing covariates used in the primary analysis were Ann Arbor stage (I/II vs. III/IV), elevated lactate dehydrogenase (LDH) (LDH >upper limit of normal [ULN] vs. LDH ≤ULN), neutropenia (absolute neutrophil count <1.5 x 10^9^/L vs. ≥1.5 x 10^9^/L), and anemia (hemoglobin <10 g/dL vs. ≥10 g/dL).

In this analysis, patient cohorts included individuals who met the study eligibility criteria, were ASCT ineligible for the given therapy line, did not have double-hit/triple-hit lymphoma, had no CNS involvement in the prior therapy line, had complete data on all six covariates, and had ≥6 months’ follow-up (the 6-month follow-up rule is described in Supplementary methods), and baseline tumor assessment. To address potential bias arising from differences in baseline characteristics between patients treated in the tafasitamab plus lenalidomide versus observational cohorts, a nearest neighbor (NN) 1:1 propensity-score matching method was used to achieve a patient-level match between cohorts [29]. Given the smaller number of patients eligible for matching in the observational cohorts compared with the tafasitamab plus lenalidomide cohort, each patient in the observational arm was randomly selected for matching with one tafasitamab plus lenalidomide cohort patient, according to an estimated propensity score (ePS) (based on a greedy matching algorithm) [30]. After patient-level matching was performed, the absolute standardized difference was evaluated to compare balance in each covariate measured between cohorts. A high degree of covariate balance was deemed to have been achieved only if the absolute standardized difference was ≤0.2 for all covariates.

### Sensitivity analysis

To evaluate the reliability of the results from the main analysis, two separate sensitivity analyses were performed. As an alternative to propensity score-based matching, we adopted the inverse probability of treatment weights method (IPTW) to form weights based on propensity scores, to account for differences between treatment and comparison groups [31]. See Supplementary methods for details of the IPTW analysis. To avoid large variance estimates in the results, caused by extreme weights from a minority of patients, a weight truncation approach was applied to remove patients whose IPTW weight is >20 [32]. The second sensitivity analysis was conducted using 1:1 NN matching with multiple imputation (MI) of missing propensity scores caused by incomplete baseline characteristics, to counteract the effects of potential bias arising from missing data. See Supplementary methods for further details of the analysis with MI. For both analyses, the set of nine clinical characteristics, prognostic outcome and laboratory parameters utilized in the primary analysis were used [23]: age (<70 vs. ≥70 years) [10, 14, 27], Ann Arbor stage (I/II vs. III/IV) [10, 14, 27], refractory to last therapy line (Yes vs. No) [10, 14, 27], number of prior lines of therapy (1 vs. 2/3) [10], history of primary refractoriness (Yes vs. No) [14, 27], prior ASCT (Yes vs. No) [10], elevated LDH (LDH >ULN vs. LDH ≤ULN) [33], neutropenia (absolute neutrophil count <1.5 x 10^9^/L vs. ≥1.5 x 10^9^/L) [34], and anemia (hemoglobin <10 g/dL vs. ≥10 g/dL) [34].

### Endpoints

The primary endpoint was OS, defined as time (in months) from the index date (start of a given therapy) until death due to any cause. Secondary endpoints were ORR, CR rate, DoR, and PFS. Secondary time-to-event endpoints are defined in Supplementary methods. Response assessments in the observational cohort followed the 1999, 2007, and 2014 International Working Group (IWG) response criteria [24–26]. In the tafasitamab plus lenalidomide cohort, the 2007 IWG response criteria were applied [25].

### Treatment-effect comparisons

For treatment-effect comparisons, the standard Kaplan–Meier (KM) method was used for analysis of the time-to-event endpoints OS and PFS. Hazard ratios (HRs) with 95% confidence intervals were estimated using a Cox proportional-hazards model; *p* values using the Log-rank test were reported. The ORR and CR rate were compared between matched cohorts using Fisher’s exact tests with *p* values reported. Descriptive KM statistics for DoR are presented.

## Results

### Study sites

Data from 3,454 patients at 200 individual sites were collected. From 158 sites (23 in North America, 118 in Europe, and 17 in the Asia Pacific region), data from 2,688 patients were captured using Medidata RAVE® (New York, NY) electronic case reports. From an additional 36 US sites, data from 766 patients were collected by the healthcare company Cardinal Health using the Cardinal Health electronic survey tool. Medical review of all recorded data was performed by MorphoSys AG (or a designated representative) for verification of patient eligibility, data accuracy, and medical plausibility. Patients who did not fulfil eligibility after medical review were excluded from analyses, and the reasons for exclusion were documented. Additionally, medical review of pertinent data such as prior therapy details, responses assessments, reasons for ASCT ineligibility, and check of baseline covariates was performed.

### Patient disposition

**Fig. 1:**
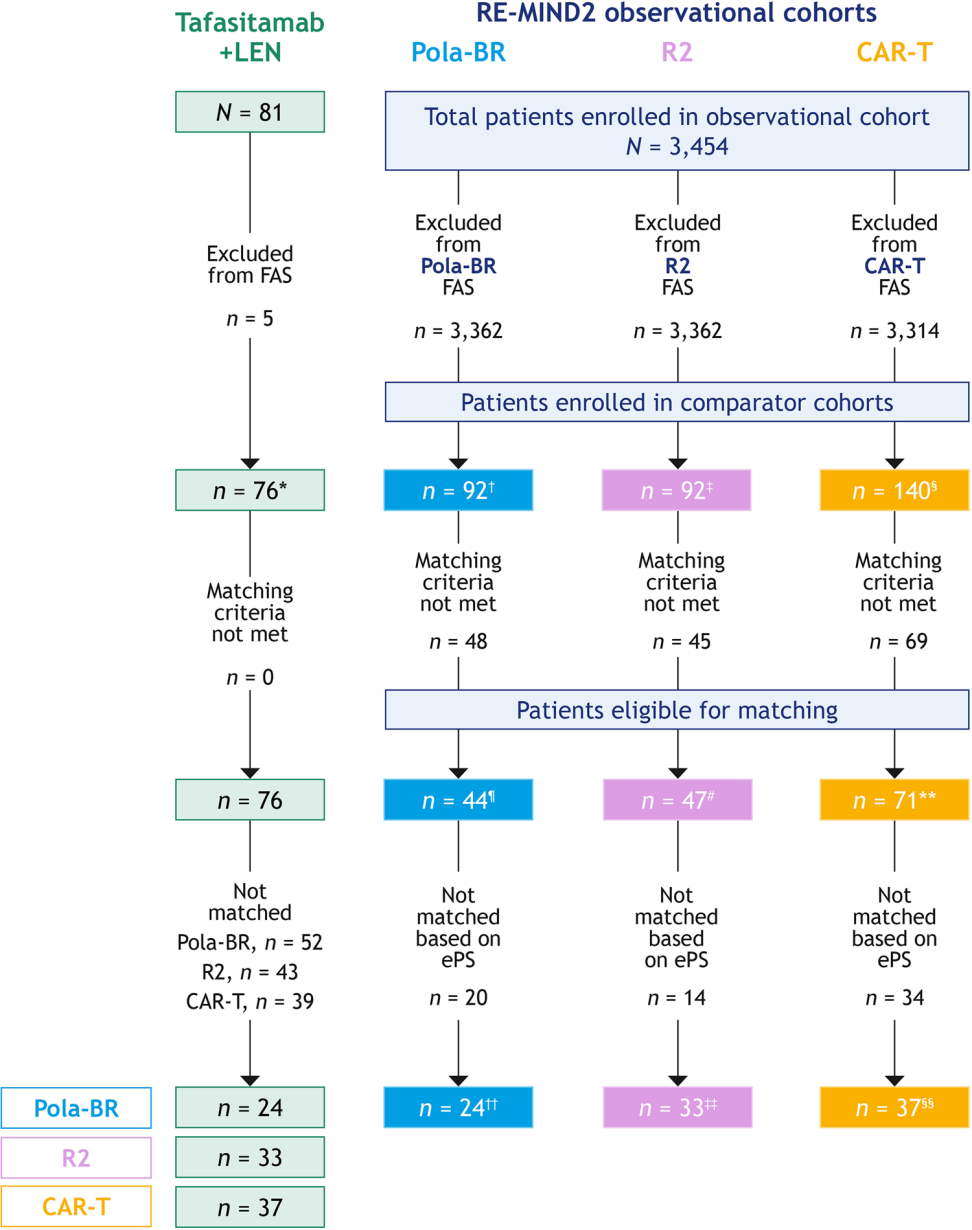
Flow of enrolled patients into matched analysis sets for the tafasitamab plus lenalidomide, pola-BR, R2, and CAR-T therapies. Abbreviations: *CAR-T*, CD19 chimeric antigen receptor T-cell therapy; *ePS*, estimated propensity score; *FAS*, full analysis set of RE-MIND2-eligible patients with a minimum of 6 months’ follow-up; *LEN*, lenalidomide; *pola-BR*, polatuzumab vedotin + bendamustine + rituximab; *R2*, rituximab + lenalidomide. *Included patients who met the eligibility/non-eligibility criteria of RE-MIND2 and who received at least one dose of tafasitamab and one dose of LEN; all patients had a minimum of 6 months’ follow-up. ^†^Included patients who met the eligibility/non-eligibility criteria of RE-MIND2 and received pola-BR; all patients had a minimum of 6 months’ follow-up. ^‡^Included patients who met the eligible/non-eligible criteria of RE-MIND2 and received R2; all patients had a minimum of 6 months’ follow-up. ^§^Included patients who met the eligibility/non-eligibility criteria of RE-MIND2 and received CAR-T; all patients had a minimum of 6 months’ follow-up. ^¶^Included a subset of enrolled patients who received pola-BR and were eligible for matching. ^#^Included a subset of enrolled patients who received R2 and were eligible for matching. **Included a subset of enrolled patients who received CAR-T and were eligible for matching. ^††^Included patients who received pola-BR and were matched 1:1 with patients from L-MIND based on ePS. ^‡‡^Included patients who received R2 and were matched 1:1 with patients from L-MIND based on ePS. ^§§^Included patients who received CAR-T and were matched 1:1 with patients from L-MIND based on ePS

In total, 3,454 and 81 patients were enrolled in the observational and the tafasitamab plus lenalidomide cohorts, respectively. After applying the eligibility and matching criteria, 44, 47, and 71 patients treated with pola-BR, R2, and CAR-T therapies, respectively, were eligible for matching from the observational cohort (Fig. 1). Reasons for exclusion from the observational cohort analysis sets included: the treatment of interest was not administered, no baseline tumor assessment, 6 months’ follow-up data were unavailable. The reasons that patients did not meet the matching criteria included: incomplete information for matching covariates, prior CNS involvement and transplant eligibility (Online Resource Table 1). Following ePS based 1:1 NN matching with patients from the tafasitamab plus lenalidomide cohort, 24, 33, and 37 patient-level matched pairs were obtained for the pola-BR, R2, and CAR-T cohorts, respectively. A high degree of covariate balance was achieved across matched analysis sets. In the pola-BR matched analysis set, perfect balance was attained; the absolute standardized difference was 0 for each matched covariate. For the matched analysis sets for R2 and CAR-T, the range of the absolute standardized difference was 0.0–0.18 and 0.0–0.19, respectively. The baseline covariates and demographic characteristics for the matched analysis sets are presented in Table 1.

**Table 1:** Demographics and baseline characteristics for the matched analyses sets for pola-BR, R2, and CAR-T therapies with the absolute standardized difference indicated for the covariates used for 1:1 NN matching

		MAS for pola-BR			MAS for R2			MAS for CAR-T		
		Tafasitamab + lenalidomide (*n* = 24)	Pola-BR (*n* = 24)	Absolute standardized difference	Tafasitamab + lenalidomide (*n* = 33)	R2 (*n* = 33)	Absolute standardized difference	Tafasitamab + lenalidomide (*n* = 37)	CAR-T (*n* = 37)	Absolute standardized difference
Matching covariates for main analysis										
Age, *n* (%)	Age <70 years	6 (25.0)	6 (25.0)	0.00	16 (48.5)	17 (51.5)	0.06	23 (62.2)	25 (67.6)	0.11
	Age ≥70 years	18 (75.0)	18 (75.0)		17 (51.5)	16 (48.5)		14 (37.8)	12 (32.4)	
Refractoriness to last therapy line, *n* (%)	Yes	17 (70.8)	17 (70.8)	0.00	19 (57.6)	21 (63.6)	0.12	23 (62.2)	23 (62.2)	0.00
	No	7 (29.2)	7 (29.2)		14 (42.4)	12 (36.4)		14 (37.8)	14 (37.8)	
Number of prior lines of therapy, *n* (%)	1	8 (33.3)	8 (33.3)	0.00	12 (36.4)	13 (39.4)	0.06	7 (18.9)	7 (18.9)	0.00
	2/3	16 (66.7)	16 (66.7)		21 (63.6)	20 (60.6)		30 (81.1)	30 (81.1)	
History of primary refractoriness, *n* (%)	Yes	8 (33.3)	8 (33.3)	0.00	14 (42.4)	17 (51.5)	0.18	10 (27.0)	13 (35.1)	0.18
	No	16 (66.7)	16 (66.7)		19 (57.6)	16 (48.5)		27 (73.0)	24 (64.9)	
Prior ASCT, *n* (%)	Yes	0 (0.0)	0 (0.0)	NE^a^	9 (27.3)	7 (21.2)	0.14	8 (21.6)	11 (29.7)	0.19
	No	24 (100)	24 (100)		24 (72.7)	26 (78.8)		29 (78.4)	26 (70.3)	
ECOG PS	0–1	22 (91.7)	22 (91.7)	0.00	27 (81.8)	27 (81.8)	0.00	34 (91.9)	34 (91.9)	0.00
	≥2	2 (8.3)	2 (8.3)		6 (18.2)	6 (18.2)		3 (8.1)	3 (8.1)	
Other characteristics										
Sex, *n* (%)	Female	10 (41.7)	10 (41.7)		17 (51.5)	11 (33.3)		18 (48.6)	17 (45.9)	
	Male	14 (58.3)	14 (58.3)		16 (48.5)	22 (66.7)		19 (51.4)	20 (54.1)	
Age at index date, years	Mean (SD)	72.3 (8.19)	73.7 (13.66)		67.9 (10.54)	69.9 (11.80)		65.8 (10.79)	63.7 (11.45)	
	Median (Q1–Q3)	73.0 (69.5–78.5)	78.5(69.5–81.0)		72.0 (58.0–75.0)	69.0 (63.0–78.0)		68.0 (58.0–75.0)	64.0 (57.0–70.0)	
	Range (min–max)	55–86	30–91		47–82	31–91		41–82	30–92	
Neutropenia (cut-off <1.5 x 10^9^/L), *n* (%)	Yes	0 (0.0)	3 (12.5)		0 (0.0)	4 (12.1)		0 (0.0)	1 (2.7)	
	No	24 (100)	17 (70.8)		33 (100)	28 (84.8)		37 (100)	36 (97.3)	
	Missing	0 (0.0)	4 (16.7)		0 (0.0)	1 (3.0)		0 (0.0)	0 (0.0)	
Anemia (cut-off hemoglobin <10 g/dL), *n* (%)	Yes	1 (4.2)	6 (25.0)		2 (6.1)	6 (18.2)		5 (13.5)	8 (21.6)	
	No	23 (95.8)	17 (70.8)		31 (93.9)	26 (78.8)		32 (86.5)	29 (78.4)	
	Missing	0 (0.0)	1 (4.2)		0 (0.0)	1 (3.0)		0 (0.0)	0 (0.0)	
Elevated LDH (>ULN), *n* (%)	Yes	14 (58.3)	18 (75.0)		20 (60.6)	22 (66.7)		19 (51.4)	21 (56.8)	
	No	10 (41.7)	4 (16.7)		13 (39.4)	8 (24.2)		18 (48.6)	15 (40.5)	
	Missing	0 (0.0)	2 (8.3)		0 (0.0)	3 (9.1)		0 (0.0)	1 (2.7)	
Race, *n* (%)	Black or African American	0 (0.0)	1 (4.2)		0 (0.0)	2 (6.1)		0 (0.0)	6 (16.2)	
	American Indian	0 (0.0)	1 (4.2)		0 (0.0)	0 (0.0)		0 (0.0)	0 (0.0)	
	Asian	0 (0.0)	0 (0.0)		0 (0.0)	2 (6.1)		0 (0.0)	2 (5.4)	
	Native Hawaiian or other Pacific Islander	0 (0.0)	0 (0.0)		0 (0.0)	0 (0.0)		0 (0.0)	0 (0.0)	
	White	23 (95.8)	19 (79.2)		28 (84.8)	17 (51.5)		34 (91.9)	19 (51.4)	
	Unknown	0 (0.0)	2 (8.3)		0 (0.0)	3 (9.1)		0 (0.0)	3 (8.1)	
	Other	0 (0.0)	1 (4.2)		1 (3.0)	9 (27.3)		0 (0.0)	7 (18.9)	
	Missing	1 (4.2)	0 (0.0)		4 (12.1)	0 (0.0)		3 (8.1)	0 (0.0)	
Primary progressive disease, *n* (%)	Yes	1 (4.2)	3 (12.5)		2 (6.1)	8 (24.2)		2 (5.4)	6 (16.2)	
	No	23 (95.8)	21 (87.5)		31 (93.9)	25 (75.8)		35 (94.6)	31 (83.8)	
Early relapse, *n* (%)	Yes	7 (29.2)	5 (20.8)		12 (36.4)	9 (27.3)		8 (21.6)	7 (18.9)	
	No	17 (70.8)	19 (79.2)		21 (63.6)	24 (72.7)		29 (78.4)	30 (81.1)	
IPI score, *n* (%)	0–2	11 (45.8)	5 (20.8)		15 (45.5)	4 (12.1)		18 (48.6)	17 (45.9)	
	3–5	13 (54.2)	14 (58.3)		18 (54.5)	16 (48.5)		19 (51.4)	13 (35.1)	
	Missing	0	5 (20.8)		0	13 (39.4)		0	7 (18.9)	
Ann Arbor disease stage, *n* (%)	I+II	3 (12.5)	4 (16.7)		8 (24.2)	2 (6.1)		6 (16.2)	8 (21.6)	
	III+IV	21 (87.5)	14 (58.3)		25 (75.8)	17 (51.5)		31 (83.8)	18 (48.6)	
	Missing	0	6 (25.0)		0	14 (42.4)		0	11 (29.7)	

^a^The absolute standardized difference is not evaluable when a binary covariate has a sole outcome; this also indicates a complete balance of the covariate. Abbreviations: *ASCT* autologous stem cell transplant, *CAR-T* CD19 chimeric antigen receptor T-cell therapy, *ECOG PS* Eastern Cooperative Oncology Group performance status, *IPI* International Prognostic Index, *LDH* lactate dehydrogenase, *MAS* matched analysis set, *NE* not evaluable, *NN* nearest neighbor, *pola-BR* polatuzumab vedotin + bendamustine + rituximab, *R2* rituximab + lenalidomide, *Q1* lower quartile; *Q3* upper quartile, *SD* standard deviation, *ULN* upper limit of normal.

### Outcomes: primary endpoint

#### Tafasitamab plus lenalidomide versus pola-BR

**Fig. 2:**
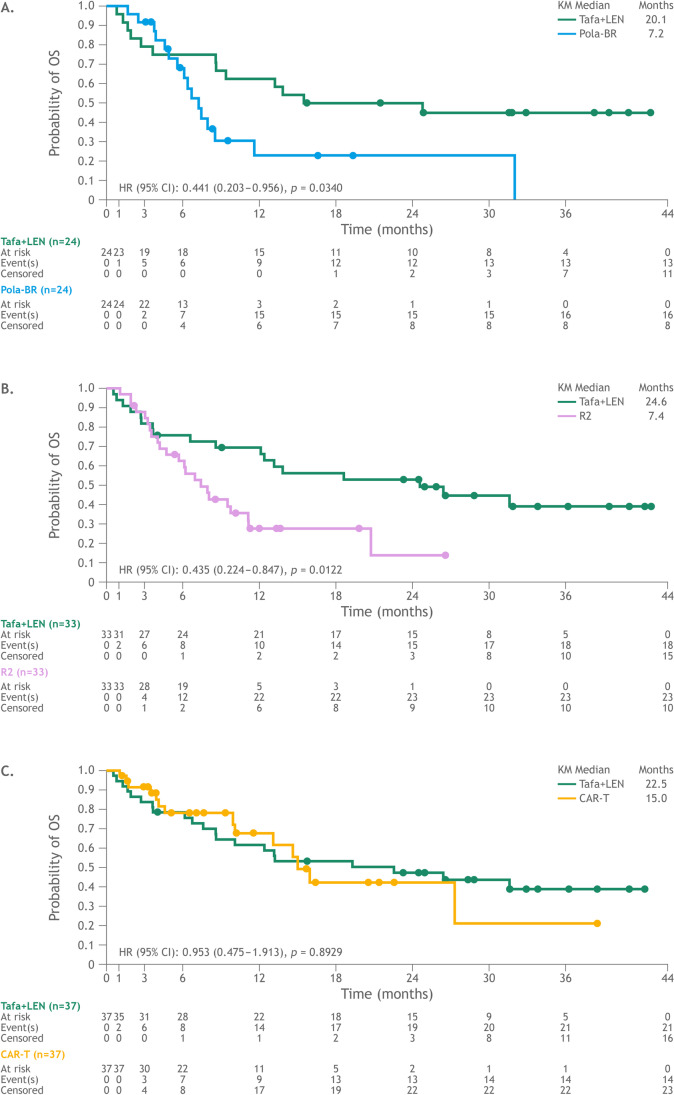
Kaplan–Meier plot of overall survival. (**A**) Tafasitamab plus lenalidomide versus pola-BR. (**B**) Tafasitamab plus lenalidomide versus R2. (**C**) Tafasitamab plus lenalidomide versus CAR-T. Abbreviations: *CAR-T*, CD19 chimeric antigen receptor T-cell therapy; *CI*, confidence interval; *HR*, hazard ratio; *KM*, Kaplan–Meier; *LEN*, lenalidomide; *OS*, overall survival; *pola-BR*, polatuzumab vedotin + bendamustine + rituximab; *R2*, rituximab + lenalidomide; *Tafa*, tafasitamab

The HR for OS favored tafasitamab plus lenalidomide versus pola-BR (HR: 0.441, 95% CI: 0.203–0.956) in the matched cohorts of 24 pairs. With a median OS of 7.2 months (median follow-up [FU]: 16.6), 15 of 16 observed OS events (94%) occurred within the first 12 months of the start of therapy with pola-BR. In the matched tafasitamab plus lenalidomide cohort, 9 of 13 observed OS events (69%) occurred within the first 12 months from the start of therapy; the median OS was 20.1 months (median FU: 31.8 months) (Fig. 2; Online Resource Table 2).

#### Tafasitamab plus lenalidomide versus R2

The HR for OS favored tafasitamab plus lenalidomide versus R2 (HR: 0.435, 95% CI: 0.224–0.847) in the matched cohorts of 33 pairs. With a median OS of 7.4 months (median FU: 13.4), 22 of 23 observed OS events (96%) occurred within the first 12 months from the start of therapy with R2. In the matched tafasitamab plus lenalidomide cohort, 10 of 18 observed OS events (55%) occurred within the first 12 months from the start of therapy; median OS was 24.6 months (median FU: 31.8 months) (Fig. 2; Online Resource Table 2).

#### Tafasitamab plus lenalidomide versus CAR-T therapies

OS was comparable between tafasitamab plus lenalidomide versus CAR-T therapies (HR: 0.953, 95% CI: 0.475–1.913) in the matched cohorts of 37 pairs. With a median OS of 15.0 months (median FU: 10.2 months), 9 of 14 observed OS events (64%) occurred within the first 12 months from the start of treatment with CAR-T therapies. In the matched tafasitamab plus lenalidomide cohort, 14 of 21 observed OS events (67%) occurred within the first 12 months from the start of therapy; the median OS was 22.5 months (median FU: 31.6 months) (Fig. 2; Online Resource Table 2).

### Outcomes: secondary endpoints

**Fig. 3.**
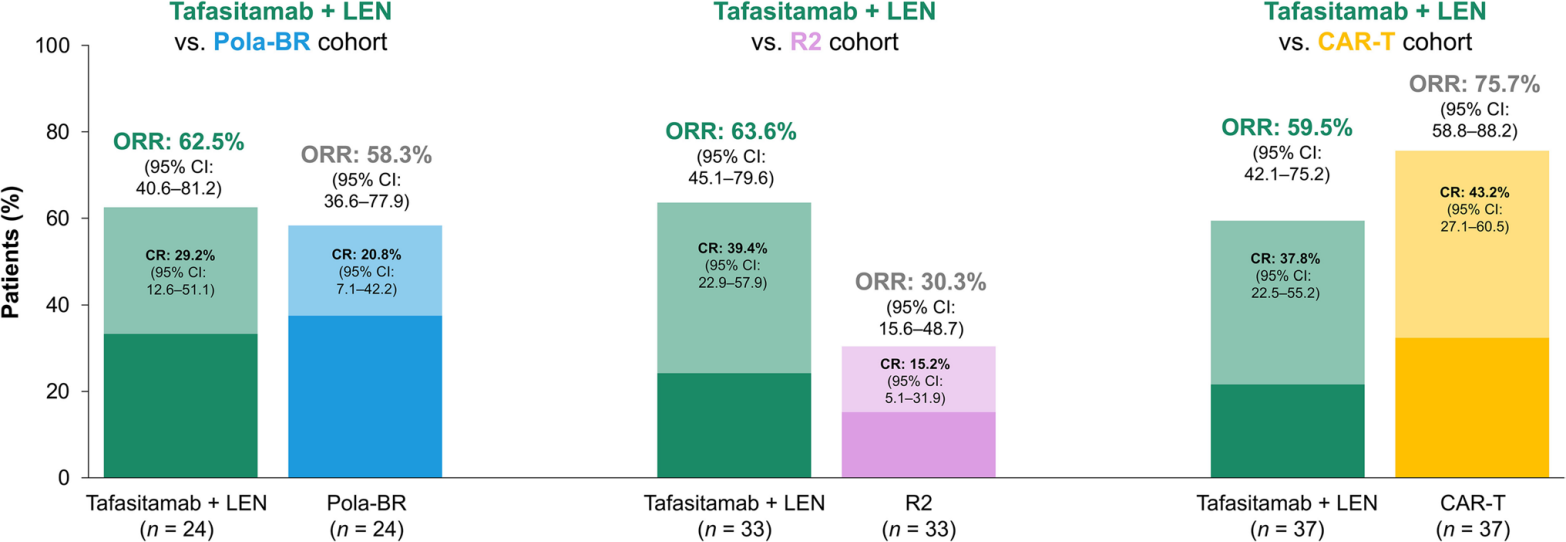
ORR and CR rate for tafasitamab plus lenalidomide versus pola-BR, R2, and CAR-T therapies. Abbreviations: *CAR-T*, CD19 chimeric antigen receptor T-cell therapy; *CI*, confidence interval; *CR*, complete response; *LEN*, lenalidomide; *ORR*, overall response rate; *pola-BR*, polatuzumab vedotin + bendamustine + rituximab; *R2*, rituximab + lenalidomide

The ORR and CR rate were not significantly higher with tafasitamab plus lenalidomide compared with pola-BR. Compared to R2, the ORR and CR rate were significantly higher with tafasitamab plus lenalidomide. Both treatment response measures were not significantly higher with CAR-T therapies versus tafasitamab plus lenalidomide (Fig. 3; Online Resource Table 2).

HR for PFS favored tafasitamab plus lenalidomide compared with pola-BR but did not reach statistical significance (HR: 0.482, 95% CI: 0.217–1.073; 24 matched pairs). With a median PFS of 5.0 months (median FU: 16.6 months), 14 of 16 observed PFS events (87%) occurred within the first 6 months from the start of therapy with pola-BR. In the matched tafasitamab plus lenalidomide cohort, 11 of 18 observed PFS events (61%) occurred within the first 6 months from the start of therapy; the median PFS was 8.0 months (median FU: 31.6 months) (Online Resource Fig. 2 and Table 2).

A significant improvement in PFS with tafasitamab plus lenalidomide, compared with R2, was detected (HR: 0.511, 95% CI: 0.281–0.927; 33 matched pairs). With a median PFS of 2.8 months (median FU: 13.4 months), 21 of 25 observed PFS events (84%) occurred within the first 6 months from the start of therapy with R2. In the matched tafasitamab plus lenalidomide cohort, 17 of 22 observed PFS events (77%) occurred within the first 6 months from the start of therapy; the median PFS was 5.9 months (median FU: 31.8 months) (Online Resource Fig. 2 and Table 2).

No significant difference in PFS with tafasitamab plus lenalidomide versus CAR-T therapies was found (HR: 0.612, 95% CI: 0.302–1.240; 37 matched pairs). With a median PFS of 4.0 months (median FU: 10.2 months), 12 of 15 observed PFS events (80%) occurred within the first 6 months from the start of therapy with CAR-T. In the matched tafasitamab plus lenalidomide cohort, 17 of 22 observed PFS events (77%) occurred within the first 6 months from the start of therapy; the median PFS was 6.3 months (median FU: 31.8 months) (Online Resource Fig. 2 and Table 2).

Descriptive analysis of DoR suggested an improvement with tafasitamab plus lenalidomide relative to the comparator treatments (Online Resource Table 2).

### Sensitivity analyses

The cohort balancing approaches used for the sensitivity analyses, with nine baseline covariates, resulted in different samples sizes in the matched analysis sets using the IPTW method. These sample sizes were: 76 patients in the tafasitamab plus lenalidomide cohorts, versus 36, 35, and 50 patients in the pola-BR, R2, and CAR-T therapies cohorts*,* respectively (Online Resource Table 3) (effective sample sizes were 72, 65, and 50 for the pola-BR, R2, and CAR-T therapies cohorts*,* respectively). One patient in the CAR-T cohort was removed due to an extreme IPTW weight. Results from the sensitivity analysis using the IPTW method supported the main analysis. HRs for OS for the comparisons of tafasitamab plus lenalidomide versus pola-BR, R2, and CAR-T therapies were 0.504 (95% CI: 0.203–1.249), 0.354 (0.171–0.731), and 0.515 (0.249–1.065), respectively (Online Resource Table 3 and Fig. 3). Results from analyses of PFS and other secondary endpoints also reinforced the main analysis (Online Resource Table 3).

For matching with multiple imputation, 63, 60, and 95 patients who received pola-BR, R2, and CAR-T therapies, respectively, were eligible. Matched analysis sets comprising 39, 41, and 39 patient pairs for pola-BR, R2, and CAR-T therapies, respectively, were established (Online Resource Table 4). Similar results were obtained for OS versus the main analysis from the sensitivity analysis using 1:1 NN matching with MI. An improvement in OS was observed with tafasitamab plus lenalidomide, compared with pola-BR (HR: 0.420, 95% CI: 0.226–0.781) and R2 (HR: 0.468, 95% CI: 0.264–0.830). A HR of 0.884 (95% CI: 0.439–1.782) was observed for the comparison between tafasitamab plus lenalidomide with CAR-T therapies (Online Resource Table 4 and Fig. 4). The results from the analysis of PFS and the other secondary endpoints aligned with those of the main analysis (Online Resource Table 4).

HRs for OS for the main analysis with six covariates, the sensitivity analyses with nine covariates using the IPTW method, and with 1:1 NN with MI of missing values are presented in Online Resource Fig. 5.

## Discussion

This extended analysis of the RE-MIND2 study was performed to evaluate the effectiveness of the tafasitamab plus lenalidomide combination relative to additional NCCN/ESMO recommended systemic therapies not included in the RE­MIND2 primary analysis. The performed analyses of outcome data built on and expand the cohort-balancing methodology of RE-MIND2, to account for a lower number of patients treated with recently approved therapies. In matched populations of patients with R/R DLBCL ineligible for ASCT, a significant and clinically meaningful improvement in OS for the tafasitamab plus lenalidomide combination, compared with the observational cohorts of pola-BR and R2, was seen. Comparable OS was observed with tafasitamab plus lenalidomide in comparison with the observational cohort of CAR-T therapies.

In an earlier publication, the RE-MIND2 primary analysis reported comparative analyses of tafasitamab plus lenalidomide versus three observational cohorts [35]. Two cohorts comprised patients who received BR or R-GemOx, and the third cohort included patients who received any systemic therapy for R/R DLBCL (the systemic therapies pooled cohort). A significant and clinically meaningful improvement in OS with tafasitamab plus lenalidomide was detected across all three matched comparisons [35]. Given the longer use of BR and R-GemOx, and the treatments included in the systemic therapies pooled cohort in routine clinical care, longer follow-up data and larger sample sizes for comparative analyses were achieved in the primary analysis (75, 74, and 76 matched patient pairs for the BR, R-GemOx, and systemic therapies pooled cohorts, respectively) [35].

In contrast, the treatments assessed in the expanded analysis are more recently approved (polatuzumab vedotin in 2019 and the CAR-T therapies tisagenlecleucel and axicabtagene ciloleucel in 2018 and 2017, respectively) [36–38]. As a result, their routine use is less prevalent, which led to fewer patients who received these treatments being available for matching in the RE-MIND2 observational cohorts. Sample sizes were therefore smaller for comparative analyses (24 and 37 patients in the pola-BR and CAR-T therapies cohorts, respectively) (Fig. 1). Although the pivotal trials assessing the R2 combination for R/R DLBCL were conducted in 2011 [39] and 2013 [8], a low number of patients were available for enrollment in the observational cohort (33 were included in comparative analyses). As the ORRs reported in both pivotal studies are ~35%, similar to the ORR for R2 in the present study (30.3%, Fig. 3), this low enrollment may reflect physician preference for regimens with improved efficacy versus R2 [8, 39]. Physicians’ choice of R2 may be reserved for selected populations (e.g., elderly patients) [39]. Furthermore, NCCN Guidelines indicate R2 as being useful in certain circumstances in the ASCT-ineligible R/R DLBCL setting (for non-germinal center B-cell-like DLBCL) as opposed to being a preferred regimen [6].

Per the observations from the primary RE-MIND2 analysis, the median OS for the comparator therapies in the expanded analysis aligns with reports in the literature [35]. The median OS observed with pola-BR in the present study (7.2 months) is similar to reports from other real-world studies of R/R DLBCL (7.7–8.3 months) [40, 41]. The median OS observed in these real-world reports is shorter than the median OS reported in the pivotal GO29365 clinical trial of pola-BR (12.4 months; *n* = 40 patients) [10], and in a recently reported extension of GO29365 (11.0 months; *n* = 106 patients) (OS was noted as not fully mature in the extension study) [42]. In contrast, median OS was not reached in a recent phase II study of pola-BR, in which median duration of follow-up was 5.4 months (range 0.7–11.9 months) [43]. Likewise, median OS reported with R2 in this analysis (7.4 months) is comparable to a report in the literature (10.7 months) [8].

Median OS with CAR-T therapies in the present study (15.0 months) is comparable to the median OS of 19.3 months reported in a recent real-world, retrospective observational study [44], whereas median OS with CAR-T therapies in pivotal clinical trials is reported as 8.3 months (*n* = 93 patients) (tisagenlecleucel) [16] and 21.1 months (*n* = 256 patients) (lisocabtagene maraleucel; not assessed in RE-MIND2) [15]. Median OS was not reached in the ZUMA-1 study of axicabtagene ciloleucel; the proportion of patients estimated as surviving to 24 months was 50.5% (*n* = 101 patients) [45]. The patients from these pivotal studies served as the reference populations for regulatory approval [13, 37, 46], and were (on average) younger [15, 16, 45]. Therefore, their overall fitness might not have precluded transplant eligibility. Conversely, patients included in the CAR-T therapies cohort in the present study were not transplant eligible. Such differences in patient characteristics may account for the overall lower performance observed in the CAR-T cohort compared with the pivotal studies. Additionally, we note that use of CAR-T therapies for second-line, non-transplant-eligible patients is not presently approved [6]. We also note that, as patients in the CAR-T therapies cohort were followed in a real-world, routine clinical setting, with less stringent tumor assessment frequency, there was a shorter duration of PFS follow-up in the CAR-T therapies versus the tafasitamab plus lenalidomide group. Additionally, CAR-T therapy was administered second-line in a small group of patients. This heterogeneity may impact the interpretation of median PFS and its HR (Online Resource Fig. 2 and Table 2). However, comparable results between tafasitamab plus lenalidomide and CAR-T therapies were observed when 3- and 6-month PFS rates (KM estimates) were examined: 69% and 52% for patients treated with tafasitamab plus lenalidomide versus 76% and 41% for patients treated with CAR-T therapies.

We note a study limitation in the duration of follow-up, inherent in an analysis of recently introduced therapies: this was shorter among patients who received pola-BR, R2, and CAR-T therapies (16.6, 13.4, and 10.2 months, respectively) versus those who received tafasitamab plus lenalidomide, during the L-MIND trial (~32 months). However, due to a high OS event rate in the observational cohort, specifically up to Month 12, a longer follow-up time would not change the conclusion of the comparative analysis of OS. While acknowledging the limitations of the short follow-up duration among the CAR-T therapy cohort in particular, and its impact represented by a wide 95% confidence interval range for median OS, we note that comparable OS was seen between the tafasitamab plus lenalidomide and CAR-T therapy cohort. Additionally, based on KM method estimation, the 6- and 12-month OS rates for patients treated with tafasitamab plus lenalidomide were 78% and 62%, respectively, similar to the OS rates at 6 (78%) and 12 (68%) months for patients treated with CAR-T therapies. A recent real-world retrospective study of CAR-T therapies indicated OS rates of 71% and 64% at 6 and 12 months using the KM method, respectively [44], further supporting the results observed in the present study. In the present analysis, it is noteworthy that the follow-up time for patients who received CAR-T was from the time patients received therapy; therefore, mortality occurring in these patients between the time of extraction and the time of infusion of T cells is not accounted for.

We acknowledge that, although outcomes for OS for the comparator therapies assessed in RE-MIND2 (e.g., for CAR-T therapies) are similar to those observed in other real-world studies, patients in the observational cohorts in our study were included to match the characteristics of patients from L-MIND. Therefore, the generalizability of our study findings is limited by the study population characteristics and may not be comparable with other studies. For example, patients who received CAR-T therapies may not typically be ASCT ineligible.

To compare the treatment effect between the tafasitamab plus lenalidomide and observational cohorts, we used propensity score-based matching and weighting for balancing clinically relevant baseline characteristics. A limitation of a non-randomized comparison is potential confounding by unknown or unmeasured baseline characteristics. However, given the magnitude of differences observed in efficacy endpoints, good balance achieved for clinically relevant baseline characteristics, and consistency with the results of multiple sensitivity analyses performed, it is unlikely that potential unmeasured confounding would impact the results to an extent that it changes the trial conclusion.

To ensure a robust analysis, several measures were adopted to assess bias and ensure that the identified observational cohorts provided an authentic comparator for the tafasitamab plus lenalidomide cohort. These were an alignment of the eligibility criteria with the L-MIND criteria and utilizing six clinically relevant baseline covariates to balance cohorts prior to analyses. As noted, the choice of six versus nine covariates used in the RE-MIND2 primary analysis balances adjustment for key clinically relevant factors, with retention of a statistically amenable sample size of matched patients to estimate response rate and time-to-event endpoints. Furthermore, to assess potential bias from patient attrition by the 1:1 matching algorithm and missing data, two sensitivity analyses were performed. In these analyses, a new set of covariates was used (nine vs. six in the main analysis), with covariates from the main analysis (i.e., ECOG PS) being replaced by other relevant prognostic and laboratory parameters (i.e., Arbor stage, LDH level, presence of neutropenia, and anemia). The benefit of the weighting method we adopted as a sensitivity analysis (the IPTW method) is its ability to leverage against patient attrition and potential selection bias to balance any population differences. The IPTW analysis confirmed the results from the main analysis (Online Resource Table 3). To account for limitations in estimating treatment effects due to missing data, we performed an additional sensitivity analysis using MI of missing data for the baseline covariates; the results from this analysis also align with those of the main analysis (Online Resource Table 4). Both sensitivity analyses served as extensions of the main analysis to provide confidence in the endpoint estimates (Online Resource Fig. 5).

## Conclusions

In summary, this expanded analysis of RE-MIND2 used information from patients in the observational cohort who received pola-BR, R2, and CAR-T therapies, and utilized multiple statistical methodologies to offset variability due to the limited patient numbers. In this retrospective comparative effectiveness analysis, a statistically significant OS difference was observed, favoring tafasitamab plus lenalidomide over pola-BR and R2 cohorts. A comparable OS was observed between the tafasitamab plus lenalidomide and the CAR-T therapies cohort. The results presented here provide physicians with further context on an increasingly broad treatment landscape.

### About Tafasitamab

Tafasitamab is a humanized Fc-modified cytolytic CD19 targeting monoclonal antibody.

In 2010, MorphoSys licensed exclusive worldwide rights to develop and commercialize tafasitamab from Xencor, Inc.

Tafasitamab incorporates an XmAb® engineered Fc domain, which mediates B-cell lysis through apoptosis and immune effector mechanisms including Antibody-Dependent Cell-Mediated Cytotoxicity (ADCC) and Antibody-Dependent Cellular Phagocytosis (ADCP).

In January 2020, MorphoSys and Incyte entered into a collaboration and licensing agreement to further develop and commercialize tafasitamab globally. Following accelerated approval by the U.S. Food and Drug Administration in July 2020, tafasitamab is being co-commercialized by MorphoSys and Incyte in the United States. Incyte has exclusive commercialization rights outside the United States.

XmAb® is a registered trademark of Xencor Inc.

## Supplementary information

ESM 1

## References

[1] Salles G, Duell J, González Barca E (2020). Tafasitamab plus lenalidomide in relapsed or refractory diffuse large B-cell lymphoma (L-MIND): a multicentre, prospective, single-arm, phase 2 study. Lancet Oncol.

[2] Duell J, Maddocks KJ, González-Barca E (2021). Long-term outcomes from the Phase II L-MIND study of tafasitamab (MOR208) plus lenalidomide in patients with relapsed or refractory diffuse large B-cell lymphoma. Haematologica.

[3] US Food & Drug Administration (2020) FDA grants accelerated approval to tafasitamab-cxix for diffuse large B-cell lymphoma. https://www.fda.gov/drugs/drug-approvals-and-databases/fda-grants-accelerated-approval-tafasitamab-cxix-diffuse-large-b-cell-lymphoma. Accessed 23 Nov 2021

[4] European Medicines Agency (2021) Tafasitamab Summary of Opinion: Committee for Medicinal Products for Human Use (CHMP). https://www.ema.europa.eu/en/documents/smop-initial/chmp-summary-positive-opinion-minjuvi_en.pdf. Accessed 27 Jul 2021

[5] Government of Canada, Health Canada, Public Affairs C and RB (2021) Drug Product Database: Minjuivi. https://health-products.canada.ca/dpd-bdpp/info.do?lang=en&code=100793. Accessed 21 Sep 2021

[6] Referenced with permission from the NCCN Clinical Practice Guidelines in Oncology (NCCN Guidelines) for B-Cell Lymphomas V.4.2023. © National Comprehensive Cancer Network, Inc. 2023. All rights reserved. Accessed June 2, 2023 or later. To view the most recent and complete version of the guideline, go online to NCCN.org. NCCN makes no warranties of any kind whatsoever regarding their content, use or application and disclaims any responsibility for their application or use in any way.

[7] Ohmachi K, Niitsu N, Uchida T (2013). Multicenter Phase II Study of Bendamustine Plus Rituximab in Patients With Relapsed or Refractory Diffuse Large B-Cell Lymphoma. J Clin Oncol.

[8] Wang M, Fowler N, Wagner-Bartak N (2013). Oral lenalidomide with rituximab in relapsed or refractory diffuse large cell, follicular and transformed lymphoma: a phase II clinical trial. Leukemia.

[9] López A, Gutiérrez A, Palacios A (2008). GEMOX-R regimen is a highly effective salvage regimen in patients with refractory/relapsing diffuse large-cell lymphoma: a phase II study. Eur J Haematol.

[10] Sehn LH, Herrera AF, Flowers CR (2019). Polatuzumab vedotin in relapsed or refractory diffuse large B-cell lymphoma. J Clin Oncol.

[11] Kalakonda N, Maerevoet M, Cavallo F (2020). Selinexor in patients with relapsed or refractory diffuse large B-cell lymphoma (SADAL): a single-arm, multinational, multicentre, open-label, phase 2 trial. Lancet Haematol.

[12] Caimi P, Ai W, Alderuccio J (2021). Loncastuximab tesirine in relapsed or refractory diffuse large B-cell lymphoma (LOTIS-2): a multicentre, open-label, single-arm, phase 2 trial. Lancet Oncol.

[13] US Food & Drug Administration (2021) FDA approves lisocabtagene maraleucel for relapsed or refractory large B-cell lymphoma. https://www.fda.gov/drugs/resources-information-approved-drugs/fda-approves-lisocabtagene-maraleucel-relapsed-or-refractory-large-b-cell-lymphoma. Accessed 8 Oct 2021

[14] Neelapu SS, Locke FL, Bartlett NL et al (2017) Axicabtagene ciloleucel CAR T-cell therapy in refractory large B-cell lymphoma. N Engl J Med 377:2531–2544. 10.1056/NEJMoa1707447 10.1056/NEJMoa1707447 PMC5882485 29226797

[15] Abramson JS, Palomba ML, Gordon LI (2020). Lisocabtagene maraleucel for patients with relapsed or refractory large B-cell lymphomas (TRANSCEND NHL 001): a multicentre seamless design study. Lancet.

[16] Schuster SJ, Bishop MR, Tam CS (2019). Tisagenlecleucel in adult relapsed or refractory diffuse large B-cell lymphoma. N Engl J Med.

[17] Tunis SR, Stryer DB, Clancy CM (2003). Practical Clinical Trials Increasing the Value of Clinical Research for Decision Making in Clinical and Health Policy. JAMA.

[18] Mullard A (2018). How much do phase III trials cost?. Nat Rev Drug Discov.

[19] US Food & Drug Administration (2016) Use of Real-World Evidence to Support Regulatory Decision-Making for Medical Devices Guidance for Industry and Food and Drug Administration Staff Preface Public Comment. http://www.fda.gov/BiologicsBloodVaccines/GuidanceComplianceRegulatoryInformation/Guida. Accessed 30 Apr 2020

[20] US Food & Drug Administration (2019) Submitting Documents Using Real-World Data and Real-World Evidence to FDA for Drugs and Biologics Guidance for Industry DRAFT GUIDANCE. https://www.fda.gov/Drugs/GuidanceComplianceRegulatoryInformation/Guidances/default.htm. Accessed 30 Apr 2020

[21] European Medicines Agency Adaptive pathways. https://www.ema.europa.eu/en/human-regulatory/research-development/adaptive-pathways. Accessed 5 Oct 2021

[22] Exley AR, Rantell K, McBlane J (2020). Clinical development of cell therapies for cancer: The regulators’ perspective. Eur J Cancer.

[23] Nowakowski GS, Yoon DH, Peters A (2022). Improved Efficacy of Tafasitamab plus Lenalidomide versus Systemic Therapies for Relapsed/Refractory DLBCL: RE-MIND2, an Observational Retrospective Matched Cohort Study. Clin Cancer Res.

[24] Cheson BD, Horning SJ, Coiffier B (1999). Report of an international workshop to standardize response criteria for non-Hodgkin’s lymphomas. J Clin Oncol.

[25] Cheson BD, Pfistner B, Juweid ME (2007). Revised response criteria for malignant lymphoma. J Clin Oncol.

[26] Cheson BD, Fisher RI, Barrington SF (2014). Recommendations for initial evaluation, staging, and response assessment of Hodgkin and non-Hodgkin lymphoma: the Lugano classification. J Clin Oncol.

[27] Crump M, Neelapu SS, Farooq U (2017). Outcomes in refractory diffuse large B-cell lymphoma: Results from the international SCHOLAR-1 study. Blood.

[28] Peng GL, Yao W-K (2017). Identification of prognostic factors in patients with diffuse large B-cell lymphoma. Indian J Pathol Microbiol.

[29] Cheng Y-J, Wang M-C (2012). Estimating Propensity Scores and Causal Survival Functions Using Prevalent Survival Data. Biometrics.

[30] Austin PC (2014). A comparison of 12 algorithms for matching on the propensity score. Stat Med.

[31] Olmos A, Govindasamy P (2015). A Practical Guide for Using Propensity Score Weighting in R. Pract Assessment, Res Eval.

[32] Franklin JM, Eddings W, Austin PC (2017). Comparing the performance of propensity score methods in healthcare database studies with rare outcomes. Stat Med.

[33] Hamlin PA, Zelenetz AD, Kewalramani T (2003). Age-adjusted International Prognostic Index predicts autologous stem cell transplantation outcome for patients with relapsed or primary refractory diffuse large B-cell lymphoma. Blood.

[34] Jurczak W, Zinzani PL, Gaidano G (2018). Phase IIa study of the CD19 antibody MOR208 in patients with relapsed or refractory B-cell non-Hodgkin’s lymphoma. Ann Oncol.

[35] Nowakowski G, Hyun Yoon D, Mondello P, et al (2021) Overall Survival with Tafasitamab + Lenalidomide (LEN) vs Routinely Administered Therapies for ASCT-Ineligible Relapsed or Refractory (R/R) Diffuse Large B-Cell Lymphoma (DLBCL): Outcomes from the Observational RE-MIND2 Study. In: Annual Meeting of the Society of Hematologic Oncology (SOHO). p ABCL-346

[36] US Food & Drug Administration (2019) FDA approves polatuzumab vedotin-piiq for diffuse large B-cell lymphoma. https://www.fda.gov/drugs/resources-information-approved-drugs/fda-approves-polatuzumab-vedotin-piiq-diffuse-large-b-cell-lymphoma. Accessed 8 Oct 2021

[37] US Food & Drug Administration (2018) FDA approves tisagenlecleucel for adults with relapsed or refractory large B-cell lymphoma. https://www.fda.gov/drugs/resources-information-approved-drugs/fda-approves-tisagenlecleucel-adults-relapsed-or-refractory-large-b-cell-lymphoma. Accessed 8 Oct 2021

[38] US Food & Drug Administration. YESCARTA (axicabtagene ciloleucel). https://www.fda.gov/vaccines-blood-biologics/cellular-gene-therapy-products/yescarta-axicabtagene-ciloleucel. Accessed 8 Oct 2021

[39] Zinzani PL, Pellegrini C, Gandolfi L (2011). Combination of lenalidomide and rituximab in elderly patients with relapsed or refractory diffuse large B-cell lymphoma: A phase 2 trial. Clin Lymphoma Myeloma Leuk.

[40] Segman Y, Ribakovsky E, Avigdor A (2020). Outcome of relapsed/refractory diffuse large B-cell lymphoma patients treated with polatuzumab vedotin-based therapy: real-life experience. Leuk Lymphoma.

[41] Northend M, Wilson W, Osborne W et al (2022) Results of a UK real world study of polatuzumab vedotin, bendamustine, and rituximab for relapsed/refractory large B-cell lymphoma. Blood Adv 6:2920–2926. 10.1182/BLOODADVANCES.2021005953 10.1182/bloodadvances.2021005953 PMC9092410 35020818

[42] Sehn LH, Hertzberg M, Opat S (2020). Polatuzumab Vedotin Plus Bendamustine and Rituximab in Relapsed/Refractory Diffuse Large B-Cell Lymphoma: Updated Results of a Phase Ib/II Randomized Study and Preliminary Results of a Single-Arm Extension. Blood.

[43] Terui Y, Rai S, Izutsu K (2021). A phase 2 study of polatuzumab vedotin + bendamustine + rituximab in relapsed/refractory diffuse large B-cell lymphoma. Cancer Sci.

[44] Sermer D, Batlevi C, Palomba ML (2020). Outcomes in patients with DLBCL treated with commercial CAR T cells compared with alternate therapies. Blood Adv.

[45] Locke FL, Ghobadi A, Jacobson CA (2019). Long-term safety and activity of axicabtagene ciloleucel in refractory large B-cell lymphoma (ZUMA-1): a single-arm, multicentre, phase 1–2 trial. Lancet Oncol.

[46] US Food & Drug Administration (2017) FDA approves axicabtagene ciloleucel for large B-cell lymphoma. https://www.fda.gov/drugs/resources-information-approved-drugs/fda-approves-axicabtagene-ciloleucel-large-b-cell-lymphoma. Accessed 9 Nov 2021

