# Correction to: RE‑MIND2: comparative effectiveness of tafasitamab plus lenalidomide versus polatuzumab vedotin/bendamustine/rituximab (pola‑BR), CAR‑T therapies, and lenalidomide/rituximab (R2) based on real‑world data in patients with relapsed/refractory diffuse large B‑cell lymphoma

**DOI:** 10.1007/s00277-023-05321-3

**Published:** 2023-07-11

**Authors:** Grzegorz S. Nowakowski, Dok Hyun Yoon, Patrizia Mondello, Erel Joffe, Anthea Peters, Isabelle Fleury, Richard Greil, Matthew Ku, Reinhard Marks, Kibum Kim, Pier Luigi Zinzani, Judith Trotman, Lorenzo Sabatelli, Eva E. Waltl, Mark Winderlich, Andrea Sporchia, Nuwan C. Kurukulasuriya, Raul Cordoba, Georg Hess, Gilles Salles

**Affiliations:** 1 Division of Hematology, Mayo Clinic, Rochester, MN USA; 2 Department of Oncology, Asan Medical Center, Songpa‑gu, Seoul, South Korea; 3 Department of Medicine, Memorial Sloan Kettering Cancer Center, New York, NY USA; 4 Department of Oncology, University of Alberta, Edmonton, AB Canada; 5 Maisonneuve‑Rosemont Hospital, Institute of Hematology, Oncology and Cell Therapy, Montreal University, Montreal, Canada; 6 Paracelsus Medical University Salzburg, Salzburg Cancer Research Institute-CCCIT, and Cancer Cluster Salzburg, Salzburg, Austria; 7 Department of Haematology, St Vincent’s Hospital and University of Melbourne, Melbourne, VIC Australia; 8 University Hospital Freiburg Internal Medicine I, Freiburg im Breisgau, Germany; 9 University of Utah, Salt Lake City, UT USA; 10 University of Illinois at Chicago, Chicago, IL USA; 11 IRCCS Azienda Ospedaliero‑Universitaria di Bologna, Istituto Di Ematologia “Seragnoli”, Bologna, Italy; 12 Dipartimento Di Medicina Specialistica, Diagnostica E Sperimentale, Universita Di Bologna, Bologna, Italy; 13 Haematology Department, Concord Repatriation General Hospital, University of Sydney, Concord, NSW Australia; 14 Incyte Biosciences International Sarl, Morges, Switzerland; 15 MorphoSys AG, Planegg, Germany; 16 MorphoSys AG, Boston, MA USA; 17 Department of Hematology, Fundacion Jimenez Diaz University Hospital, Health Research Institute IISFJD, Madrid, Spain; 18 Department of Hematology, Oncology and Pneumology, University Medical School of the Johannes Gutenberg-University Mainz, Mainz, Germany

**Correction to: Annals of Hematology**

10.1007/s00277-023-05196-4

Following the online publication of this paper*,* the authors have learned that the National Comprehensive Cancer Network (NCCN) requires some editorial changes relating to how their guidelines are referenced within the paper.

The original article has been corrected.

