# Supplementary material for: RE-MIND2: comparative effectiveness of tafasitamab plus lenalidomide versus polatuzumab vedotin/bendamustine/rituximab (pola-BR), CAR-T therapies, and lenalidomide/rituximab (R2) based on real-world data in patients with relapsed/refractory diffuse large B-cell lymphoma
Source: Ann Hematol. 2023 May 12;102(7):1773–87. doi: 10.1007/s00277-023-05196-4 (PMC10261238; doi:10.1007/s00277-023-05196-4)
Supplement: Supplementary file 1 — ESM 1 [file 277_2023_5196_MOESM1_ESM.docx]

**RE-MIND2: comparative effectiveness of tafasitamab plus lenalidomide versus polatuzumab vedotin/bendamustine/rituximab (pola-BR), CAR-T therapies, and lenalidomide/rituximab (R2) based on real-world data in patients with relapsed/refractory diffuse large B-cell lymphoma**

Author names
Grzegorz S. Nowakowski, MD, PhD^1^*, Dok Hyun Yoon, MD, PhD^2^, Patrizia Mondello, MD, PhD, MSc^3^, Erel Joffe, MD^3^, Anthea Peters, MD^4^, Isabelle Fleury, MD^5^, Richard Greil, MD^6^, Matthew Ku, MBBS, FRACP, FRCPA, PhD^7^, Reinhard Marks, MD^8^, Kibum Kim, PhD^9,10^, Pier Luigi Zinzani, MD, PhD^11,12^, Judith Trotman, FRACP^13^, Lorenzo Sabatelli, PhD^14^, Eva E. Waltl, PhD^15^, Mark Winderlich, PhD, MSc^15^, Andrea Sporchia MD^15^, Nuwan C. Kurukulasuriya, PhD^16^, Raul Cordoba^17^, Georg Hess, MD^18^, Gilles Salles, MD^3^

*Corresponding author

# Affiliations

^1^Division of Hematology, Mayo Clinic, Rochester, MN, USA

^2^Department of Oncology, Asan Medical Center, Songpa-gu, Seoul, South Korea

^3^Department of Medicine, Memorial Sloan Kettering Cancer Center, New York, NY, USA

^4^Department of Oncology, University of Alberta, Edmonton, Alberta, Canada

^5^Maisonneuve-Rosemont Hospital, Institute of Hematology, Oncology and Cell Therapy, Montreal University, Montreal, Canada

^6^Paracelsus Medical University Salzburg, Salzburg Cancer Research Institute-CCCIT, and Cancer Cluster Salzburg, Salzburg, Austria

^7^Department of Haematology, St Vincent’s Hospital and University of Melbourne, Melbourne, Victoria, Australia

^8^University Hospital Freiburg Internal Medicine I, Freiburg im Breisgau, Germany

^9^University of Utah, Salt Lake City, UT, USA

^10^University of Illinois at Chicago, Chicago, IL, USA

^11^IRCCS Azienda Ospedaliero-Universitaria di Bologna, Istituto di Ematologia “Seràgnoli”, Bologna, Italy

^12^Dipartimento di Medicina Specialistica, Diagnostica e Sperimentale Università di Bologna, Bologna, Italy

^13^Haematology Department, Concord Repatriation General Hospital, University of Sydney, Concord, NSW, Australia

^14^Incyte Biosciences International Sàrl, Morges, Switzerland

^15^MorphoSys AG, Planegg, Germany

^16^MorphoSys AG, Boston, MA, USA

^17^Department of Hematology, Fundacion Jimenez Diaz University Hospital, Health Research Institute IISFJD, Madrid, Spain

^18^Department of Hematology, Oncology and Pneumology, University Medical School of the Johannes Gutenberg-University Mainz, Germany

**Corresponding author:**

Grzegorz Nowakowski

Division of Hematology

Mayo Clinic

200 First St. SW

Rochester

Minnesota 55905

United States

Phone: +01 (507) 405-0312

Email: Nowakowski.Grzegorz@mayo.edu

# Supplementary methods

**Assigning lines of therapy in the observational cohort**

In the observational cohort, data from first to fourth therapy line were collected. The criteria for relapsing/refractory diffuse large B-cell lymphoma (R/R DLBCL) were determined by the study site investigators and validated through medical review of each patient’s first-line treatment history and statistical validation. As a result, 65 of 3,454 (1.9%) patients were excluded from analysis as R/R DLBCL status could not be confirmed.

The study database recorded details of prior treatment with ≥1 therapy lines (e.g., start/end date of treatment, best response, date of progression) for each patient in the observational cohort; if an enrolled patient received a treatment of interest in a particular line, the data for this patient’s therapy line was selected for matching and analysis.

**Definition of refractoriness to last prior treatment line**

A patient is considered to be refractory to the last prior line if any one of the following criteria is met:

(1) Disease progression during the last prior line treatment (systemic anti-DLBCL medication). That is, the status at the end of treatment is progressive disease (PD)

(2) Best response of less than partial response (PR) (i.e., PD or stable disease [SD]) in the last prior line (in the context of refractoriness, ‘best response’ refers to the best assessment result out of all tumor assessments for L-MIND cohort, whereas for the observation cohort, ‘best response’ refers to the best assessment result out of ‘initial response’, ‘best response’ and ‘disease status at discontinuation’ of the last prior line treatment)

(3) Reason for discontinuation of last prior therapy is disease progression

(4) Disease progression within ≤6 months (183 days) from the completion of the last prior treatment line. The ‘end date’ of the last prior treatment line as entered in the electronic case report form (eCRF) will be considered as date of completion. If multiple end dates are given (e.g., for combination treatment), the end date for the drug that has been discontinued latest will be used

(5) Treatment of the given line started within ≤6 months (183 days) from the completion of the last prior treatment line.

**Definition of primary refractoriness**

A patient will be considered as primary refractory if any one of the following criteria are met:

(1) Disease progression during the first-line treatment (systemic anti-DLBCL medication). That is, the status at the end of treatment is PD

(2) Best response (as per section 1.1.2) of less than PR (i.e., PD or SD) in the first-line treatment

(3) Reason for discontinuation of first-line treatment is disease progression

(4) Disease progression within ≤6 months (183 days) from the completion of first-line therapy. The ‘end date’ of the first-line treatment as entered in the eCRF will be considered as date of completion. If multiple end dates are given (e.g., for combination treatment), the end date for the drug that has been discontinued latest will be used

(5) Treatment of the second therapy line started within ≤6 months (183 days) from the completion of the first line of therapy

**The 6-month follow-up rule**

To address potential bias in favor of the tafasitamab plus lenalidomide cohort, in which responses to treatment may have been more effectively captured in the L-MIND clinical trial setting, a minimum of 6 months’ follow-up for the comparative analysis was applied. This avoided overestimating the rate of non-responders in the observational cohort.

*1.5 Definitions of secondary time-to-event endpoints*

Duration of response was defined as the elapsed time (in months) between the date of first documented response for a given therapy, and the date of event defined as the first documented progression or death. Progression-free survival was defined as time (in months) from the index date for a given therapy to the date of first documented tumor progression or death.

**Sensitivity analysis using the inverse probability of treatment weights method (IPTW)**

When using the IPTW, each individual in the cohort is assigned a weight based on an estimated propensity score (ePS). Applying this weight when conducting statistical tests reduces or removes the impact of confounders. The IPTW approach uses the entire cohort of patients eligible for cohort balancing and can address a larger number of confounding variables.^1^ In the present study, IPTW was used to estimate the average treatment effect in the treated population by executing the following steps: (1) to obtain the propensity score ($p_{i})$of each patient using nine baseline covariates in the logistic regression model. (2) To calculate the weight of patients in both cohorts. The weight of a patient in the L-MIND cohort is 1, whereas the weight of a patient in the observational cohort (denoted as $w\left( c \right)$) is estimated by:

$$w\left( c \right)=\frac{p_{i}}{1-p_{i}}$$

(3) To avoid large variance estimates in the results, caused by extreme weights from a minority of patients, patients with weights >30 were removed. (4) The treatment effect was then estimated by using the weighted approach. Weights were employed using WEIGHT options in the relevant SAS analysis procedures.

**Sensitivity analysis using 1:1 nearest neighbor (NN) matching with multiple imputation (MI) of missing values**

The missing ePS for each patient was obtained from a mean of 30 imputed datasets based on Rubin’s approach.^2^ This consisted of three phases: (1) imputation phase: the fully conditional specification option in PROC MI in SAS 9.4 will be used to generate m = 30 imputed datasets. Missing patterns will be assessed. The process assumes a missing at random pattern of missingness. (2) Analysis phase: ePS will be calculated using a logistic regression model with nine baseline covariates (see section 2.7) as covariates for each imputed data set. (3) Pooling phase: the results from the analysis of each imputed dataset will be combined for overall inference using Rubin’s rules, which account for the uncertainty associated with the imputed value, using SAS PROC MIANALYZE. The pooled ePS thus obtained will then be used to repeat the NN 1:1 matching.

**References**

1. Austin PC, Stuart EA. The performance of inverse probability of treatment weighting and full matching on the propensity score in the presence of model misspecification when estimating the effect of treatment on survival outcomes. Stat Methods Med Res 2017;26(4):1654.

2. Rubin DB. An overview of multiple imputation. In: Proceedings of the Survey Research Methods Section of the American Statistical Association. American Statistical Association, 1988:79–84.

# Supplementary figures

## **Fig. 1 RE-MIND2 study design indicating assessment periods.** Patients who received at least two therapy lines for DLBCL were assigned an index date (index date 2L, 3L, or 4L) for each eligible therapy line. Pre-index period: time between initial DLBCL diagnosis and index date of treatments (2L, 3L, or 4L). Index date: start of R/R DLBCL treatment (2L, 3L, or 4L). Observational period: time between index date and end of follow-up including survival assessment. Baseline: 28 days of baseline assessment prior to index date. Abbreviations: *DLBCL*, diffuse large B-cell lymphoma; *L*, therapy line; *R/R*, relapsed/refractory.


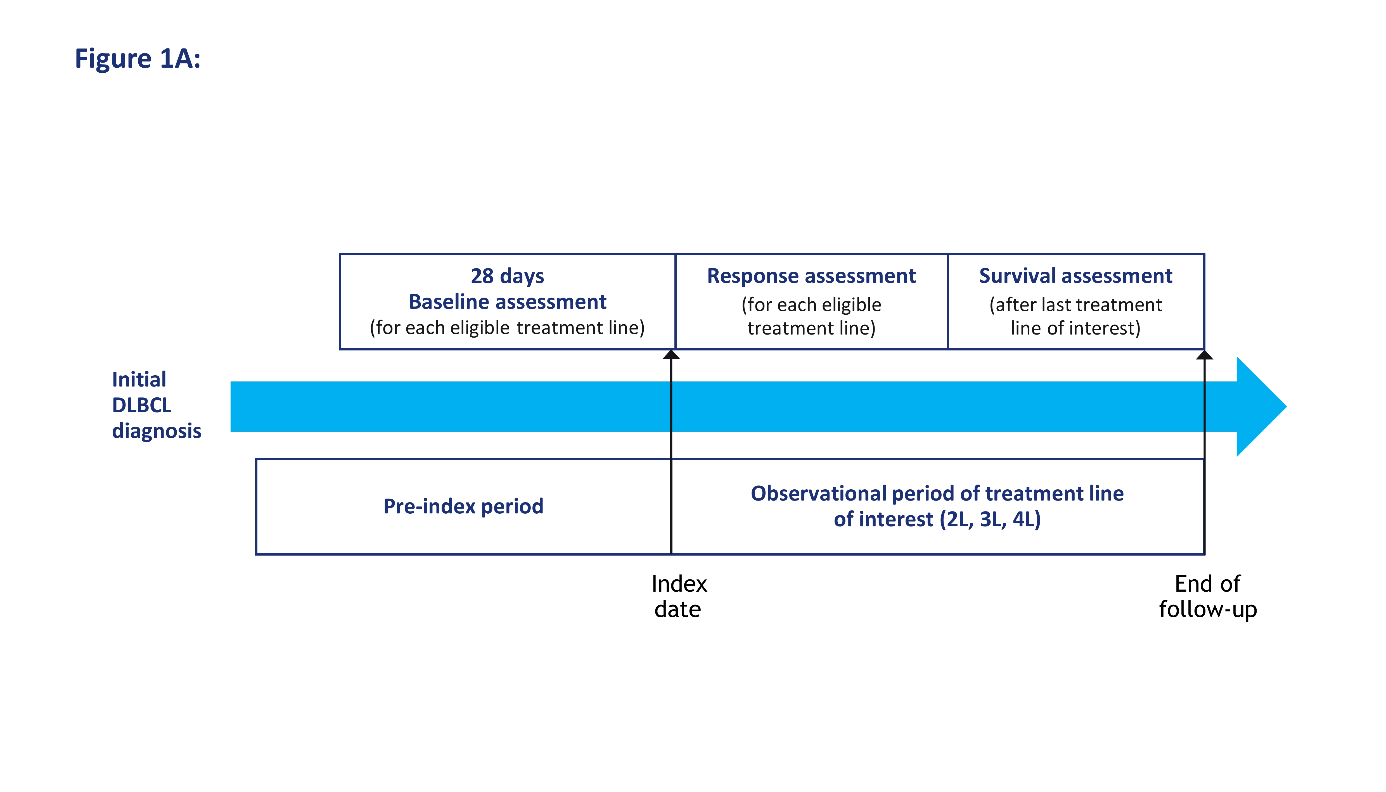


## Fig. 2 Kaplan–Meier plot of PFS. (A) Tafasitamab plus lenalidomide versus pola-BR. (B) Tafasitamab plus lenalidomide versus R2. (C) Tafasitamab plus lenalidomide versus CAR-T. Abbreviations: *CAR-T*, CD19 chimeric antigen receptor T-cell therapy; *CI*, confidence interval; *HR*, hazard ratio; *KM*, Kaplan–Meier; *LEN*, lenalidomide; *PFS*, progression-free survival; *pola-BR*, polatuzumab vedotin + bendamustine rituximab; *R2*, rituximab + lenalidomide; *Tafa*, tafasitamab.


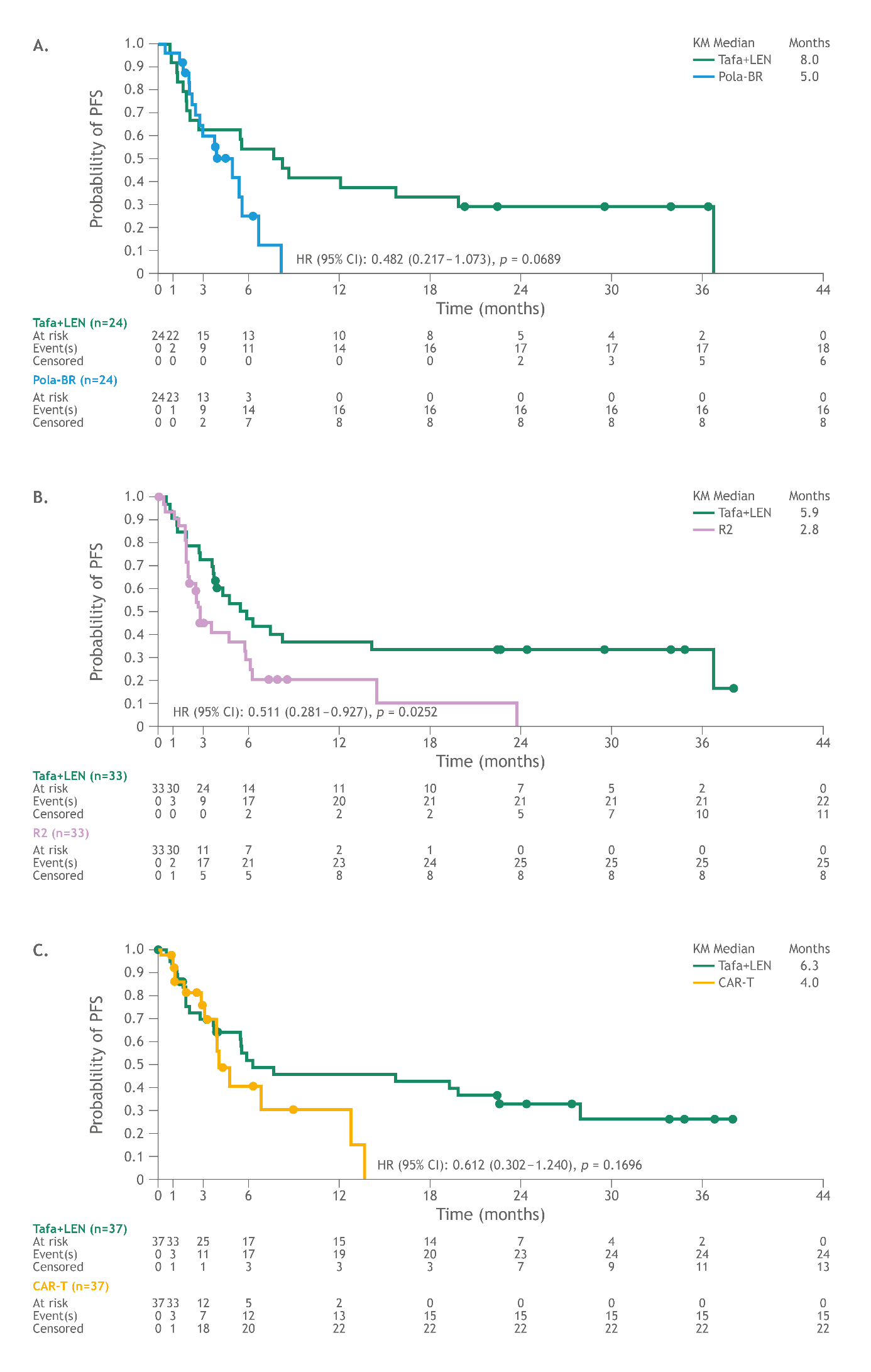


## Fig. **3** Kaplan–Meier plot of OS using the IPTW method balanced on nine baseline covariates**.** (A) Tafasitamab plus lenalidomide versus pola-BR. (B) Tafasitamab plus lenalidomide versus R2. (C) Tafasitamab plus lenalidomide versus CAR-T. KM median, hazard ratio, log-rank test *p* value and effective sample size shown at the bottom of KM curves are weighted using the IPTW method. Abbreviations: *CAR-T*, CD19 chimeric antigen receptor T-cell therapy; *CI*, confidence interval; *HR*, hazard ratio; *IPTW*, inverse probability of treatment weighting; *KM*, Kaplan–Meier; *LEN*, lenalidomide; *pola-BR*, polatuzumab vedotin + bendamustine + rituximab; *OS*, overall survival; *R2*, rituximab + lenalidomide; *Tafa*, tafasitamab.


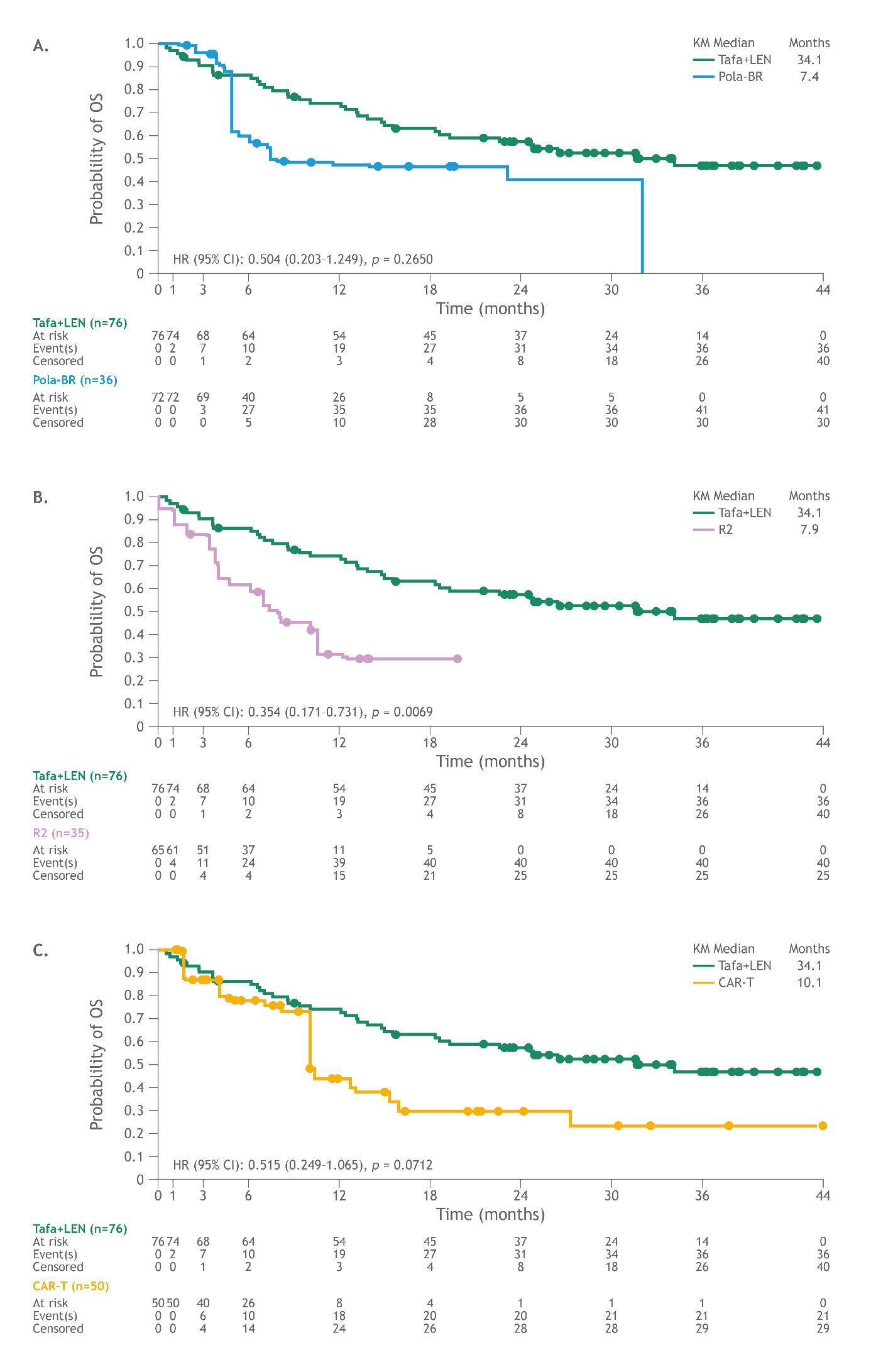


## Fig. **4** Kaplan–Meier plot of OS **using** the matched analysis set balanced on nine baseline covariates after multiple imputation. (A) Tafasitamab plus lenalidomide versus pola-BR. (B) Tafasitamab plus lenalidomide versus R2. (C) Tafasitamab plus lenalidomide versus CAR-T. Abbreviations: *CAR-T*, CD19 chimeric antigen receptor T-cell therapy; *CI*, confidence interval; *HR*, hazard ratio; *KM*, Kaplan–Meier; *LEN*, lenalidomide; *OS*, overall survival; *pola-BR*, polatuzumab vedotin + bendamustine + rituximab; *R2*, rituximab + lenalidomide; *Tafa*, tafasitamab.


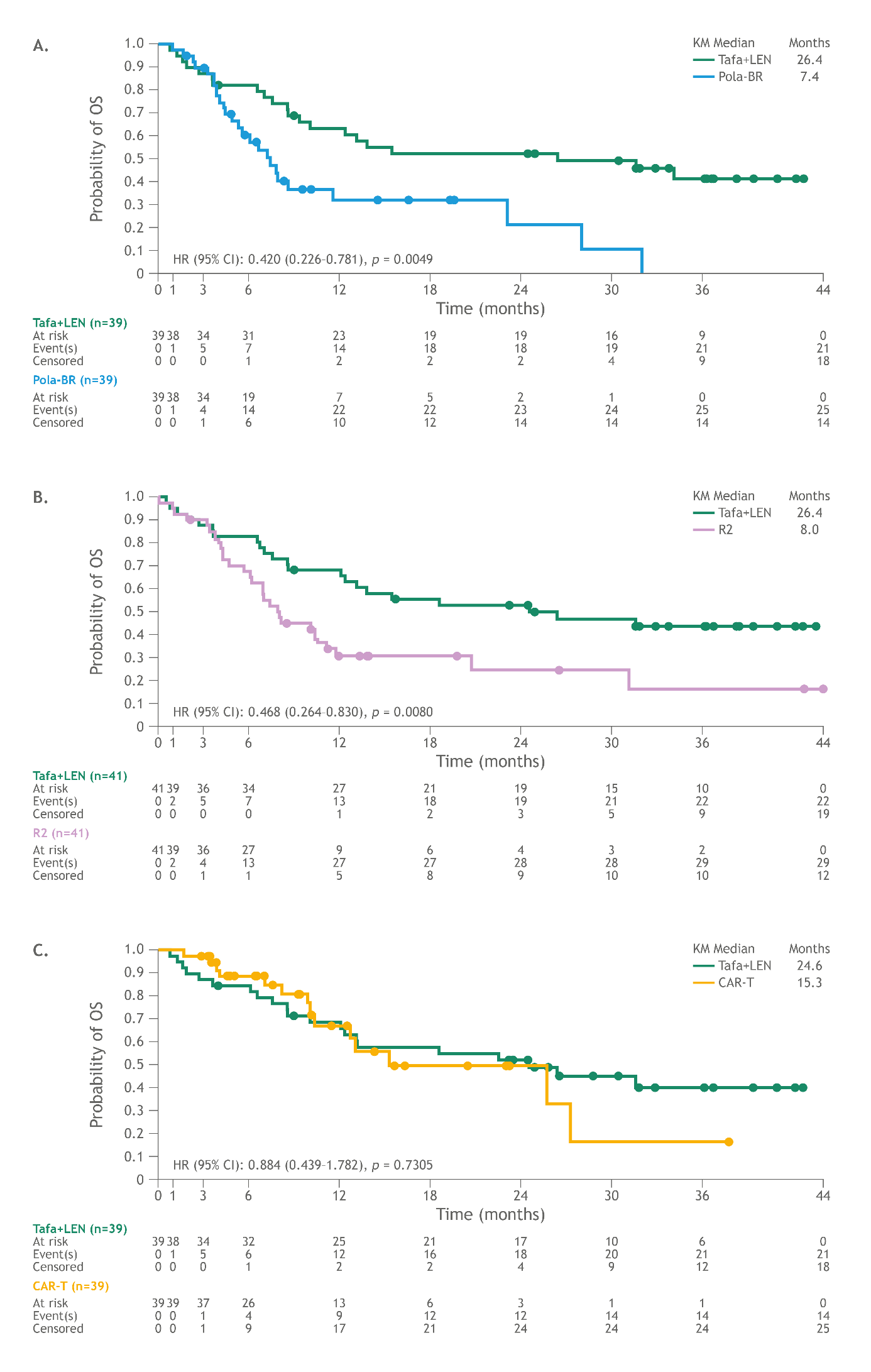


## Fig. 5 Forest plots depicting OS for tafasitamab plus lenalidomide versus (A) pola-BR, (B) R2, and (C) CAR-T therapies for the main analysis with six covariates, the sensitivity analyses with nine covariates using the IPTW method, and with 1:1 NN with MI of missing values. A hazard ratio <1 indicates a superior treatment effect of tafasitamab plus lenalidomide. Abbreviations: *CAR-T*, chimeric antigen receptor T-cell therapy; *CI*, confidence interval; *E/E*, number of events in the tafasitamab plus lenalidomide/observational cohort; *FAS*, full analysis set; *FAS_elig_9cov_IPTW*, FAS of patients eligible for matching with nine baseline covariates using the IPTW method; *HR*, hazard ratio; *IPTW*, inverse probability of treatment weights method; *LEN*, lenalidomide; *MAS*, matched analysis set; *MI*, multiple imputation; *NN*, nearest neighbor; *N/N*, number of patients in the tafasitamab plus lenalidomide cohort/number of patients in the observational cohort; *OS*, overall survival; *pola-BR*, polatuzumab vedotin + bendamustine + rituximab; *R2*, rituximab + lenalidomide; *Tafa*, tafasitamab.


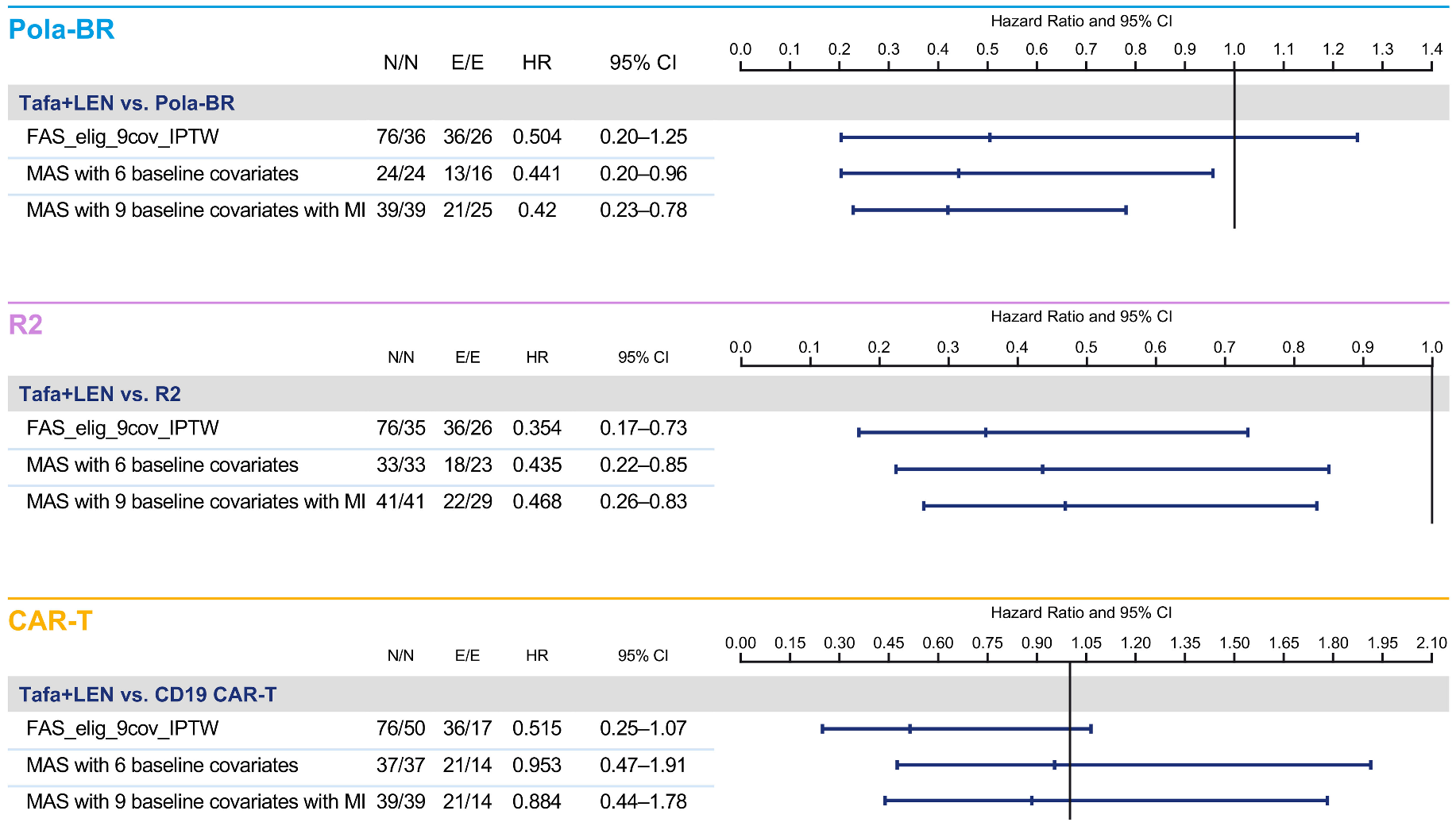


# Supplementary tables

## Table 1 Reasons for patient attrition in observational cohorts for treatments of interest.

| **Exclusion reason from observational cohort FAS** | **Pola-BR** | **R2** | **CAR-T** |
| --- | --- | --- | --- |
| Total patients excluded from FAS | 3,362 (97.3) | 3,362 (97.3) | 3,314 (95.9) |
| Duplicate patients, n (%) | 8 (0.2) | 8 (0.2) | 8 (0.2) |
| R/R DLBCL could not be confirmed, n (%) | 65 (1.9) | 65 (1.9) | 65 (1.9) |
| No baseline tumor assessment, n (%) | 15 (0.4) | 21 (0.6) | 39 (1.1) |
| Treatment of interest was not administered, n (%) | 3,260 (94.4) | 3,254 (94.2) | 3,193 (92.4) |
| 6 months‘ follow-up data was not available, n (%) | 14 (0.4) | 14 (0.4) | 9 (0.3) |
| **Reason for not meeting matching criteria** | | | |
| Total patients not meeting matching criteria | 48 (15.8) | 45 (14.9) | 69 (22.8) |
| Having double-hit/triple-hit lymphoma | 18 (5.9) | 14 (4.6) | 20 (6.6) |
| Transplant-eligible patients | 7 (2.3) | 6 (2.0) | 20 (6.6) |
| Prior CNS involvement | 4 (1.3) | 12 (4.0) | 5 (1.7) |
| Incomplete information for matching covariates | 19 (6.3) | 13 (4.3) | 24 (7.9) |

Abbreviations: *CAR-T*, CD19 chimeric antigen receptor T-cell therapy; *CNS*, central nervous system; *DLBCL*, diffuse large B-cell lymphoma; *FAS*, full analysis set; *pola-BR*, polatuzumab vedotin + bendamustine + rituximab; *R2*, rituximab + lenalidomide; *R/R*, relapsed/refractory.

## Table 2 **Comparative analysis results for primary and secondary endpoints (descriptive statistics for DoR) for tafasitamab plus lenalidomide versus pola-BR, R2, and CAR‑T therapies.**

|  | **MAS for pola-BR** | | **MAS for R2** | | **MAS for CAR-T** | |
| --- | --- | --- | --- | --- | --- | --- |
|  | **Tafasitamab + lenalidomide (*n =* 24)** | **Pola-BR**  **(*n* =** **24)** | **Tafasitamab + lenalidomide (*n* = 33)** | **R2**  **(*n* =** **33)** | **Tafasitamab + lenalidomide (*n* =** **37)** | **CAR-T**  **(*n* =** **37)** |
| **Median OS**, mo  (95% CI) | 20.1 (8.6–NR) | 7.2 (4.9–11.6) | 24.6 (12.1–NR) | 7.4 (4.2–11.1) | 22.5 (8.6–NR) | 15.0 (10.1–NR) |
| HR for OS  (95% CI) *p* value* | 0.441 (0.203–0.956) 0.0340 | | 0.435 (0.224–0.847) 0.0122 | | 0.953 (0.475–1.913) 0.8929 | |
| **ORR**, *n* (%)  (95% CI) *p* value^†^ | 15 (62.5)  (40.6–81.2) | 14 (58.3)  (36.6–77.9) | 21 (63.6)  (45.1–79.6) | 10 (30.3)  (15.6–48.7) | 22 (59.5)  (42.1–75.2) | 28 (75.7)  (58.8–88.2) |
|  | 1.0000 | | 0.0130 | | 0.2140 | |
| **Difference of ORR**, (%) (95% CI)  *p* value^‡^ | 4.17 (-25.626–33.427)  1.0000 | | 33.33  (7.972–55.594) 0.0130 | | -16.22  (-38.970–7.877) 0.2140 | |
| **CR rate as best response**, *n* (%)  (95% CI) *p* value^†^ | 7 (29.2)  (12.6–51.1) | 5 (20.8)  (7.1–42.2) | 13 (39.4)  (22.9–57.9) | 5 (15.2)  (5.1–31.9) | 14 (37.8)  (22.5–55.2) | 16 (43.2)  (27.1–60.5) |
|  | 0.7400 | | 0.0514 | | 0.8131 | |
| **Difference of CR rate**, (%)  (95% CI)  *p* value^‡^ | 8.33  (-21.656–37.257)  0.7400 | | 24.24  (-1.329–47.563)  0.0514 | | -5.41  (-28.865–18.500)  0.8131 | |
| **Median PFS,** mo (95% CI) | 8.0 (1.9–19.9) | 5.0 (2.5–5.6) | 5.9 (3.6–36.7) | 2.8 (2.0–5.8) | 6.3 (3.6–22.5) | 4.0 (3.1–12.8) |
| HR  (95% CI)  *p* value* | 0.482 (0.217–1.073) 0.0689 | | 0.511 (0.281–0.927) 0.0252 | | 0.612 (0.302–1.240) 0.1696 | |
| **Median DoR**, mo  (95% CI) | 17.7 (3.6–34.8) | 2.3 (0.3–6.1) | 34.8 (3.6–34.8) | 12.4 (2.7–19.3) | 26.1 (4.4–NR) | 5.9 (2.0–10.0) |

*Calculated using Log-rank test. ^†^Calculated using Fisher’s exact test. ^‡^Calculated using Chan–Zhang method. Abbreviations: *CAR-T*, CD19 chimeric antigen receptor T-cell therapy; *CI*, confidence interval; *CR*, complete response; *DoR*, duration of response; *HR*, hazard ratio; *MAS*, matched analysis set; *mo*, months; *NR*, not reached; *ORR*, overall response rate; *OS*, overall survival; *PFS*, progression-free survival; *pola-BR*, polatuzumab vedotin + bendamustine + rituximab; *R2*, rituximab + lenalidomide.

## Table 3 **Results from sensitivity analysis of primary and secondary endpoints using IPTW method balanced on nine baseline covariates for tafasitamab plus lenalidomide versus pola-BR, R2, and CAR‑T therapies.**

|  | **Tafasitamab + lenalidomide (*n =* 76)** | **Pola-BR**  **(*n* =** **36)** | **Tafasitamab + lenalidomide (*n* = 76)** | **R2**  **(*n* =** **35)** | **Tafasitamab + lenalidomide (*n* =** **76)** | **CAR-T**  **(*n* =** **50)** |
| --- | --- | --- | --- | --- | --- | --- |
| **Median OS,** mo 95% CI | 34.1 (18.3–NR) | 7.4 (1.1–32.0) | 34.1 (18.3–NR) | 7.9 (3.8–NR) | 34.1  (18.3–NR) | 10.1 (NR–NR) |
| HR for OS  (95% CI) *p* value* | 0.504 (0.203–1.249) 0.2650 | | 0.354 (0.171–0.731) 0.0069 | | 0.515 (0.249–1.065) 0.0712 | |
| **ORR**, n (%)  (95% CI)  *p* value^†^ | 51 (67.1) (55.4–77.5) | 17 (47.2) (30.4–64.5) | 51 (67.1) (55.4–77.5) | 10 (28.6) (14.6–46.3) | 51 (67.1) (55.4–77.5) | 40 (80.0) (66.3–90.0) |
|  | 0.5607 | | 0.1109 | | 0.3893 | |
| **Difference of ORR**, (%) (95% CI)^‡^  *p* value | 12.34 (-25.642–50.323) 0.5243 | | 25.23 (-0.413–50.866) 0.0538 | | -10.23 (-34.453–14.000) 0.4081 | |
| **CR rate as best response**, *n* (%)  (95% CI) *p* value^†^ | 29 (38.2) (27.2–50.0) | 7 (19.4) (8.2–36.0) | 29 (38.2) (27.2–50.0) | 2 (5.7) (0.7–19.2) | 29 (38.2) (27.2–50.0) | 23 (46.0) (31.8–60.7) |
|  | 0.7366 | | 0.0784 | | 0.9389 | |
| **Difference of CR rate**, (%)  (95% CI)^‡^  *p* value | 7.38 (-31.680–46.430) 0.7113 | | 31.14 (14.099–48.182) 0.0003 | | -1.00 (-26.911–24.901) 0.9394 | |
| **Median PFS,** mo (95% CI) | 12.1 (5.9–22.5) | 4.5 (0.5–13.3) | 12.1  (5.9–22.5) | 4.4 (2.6–6.3) | 12.1  (5.9–22.5) | 3.9 (NR–NR) |
| HR  (95% CI)  *p* value* | 0.416 (0.229–0.754)  0.1043 | | 0.404 (0.213–0.768) 0.0179 | | 0.534 (0.244–1.168) 0.1261 | |
| **Median DoR**, mo  (95% CI) | 26.1 (13.9–NR) | 6.1 (2.1–11.3) | 26.1 (13.9–NR) | 2.7 (1.7–NR) | 26.1 (13.9–NR) | 5.9 (1.0–11.6) |

*Calculated using Log-rank test. ^†^Calculated using Chi-square test. ^‡^The differences of ORR/CR in each comparison group are estimated using IPTW method. Abbreviations: *CAR-T*, CD19 chimeric antigen receptor T-cell therapy; *CI*, confidence interval; *CR*, complete response; *DoR*, duration of response; *HR*, hazard ratio; *IPTW*, inverse probability of treatment weighting; *mo*, months; *NR*, not reached; *ORR*, overall response rate; *OS*, overall survival; *PFS*, progression-free survival; *pola-BR*, polatuzumab vedotin + bendamustine + rituximab; *R2*, rituximab + lenalidomide.

## Table 4 Results from sensitivity analysis of primary and secondary endpoints of matched analysis set balanced on nine baseline covariates after multiple imputation for tafasitamab plus lenalidomide versus pola-BR, R2, and CAR-T**.**

|  | **MAS for pola-BR** | | **MAS for R2** | | **MAS for CAR-T** | |
| --- | --- | --- | --- | --- | --- | --- |
|  | **Tafasitamab + lenalidomide (*n =* 39)** | **Pola-BR**  **(*n* =** **39)** | **Tafasitamab + lenalidomide (*n* = 41)** | **R2**  **(*n* =** **41)** | **Tafasitamab + lenalidomide (*n* =** **39)** | **CAR-T**  **(*n* =** **39)** |
| **Median OS,** mo 95% CI | 26.4 (9.4–NR) | 7.4 (4.9–11.6) | 24.6 (12.1–NR) | 8.0 (6.1–11.1) | 24.6 (12.1–NR) | 15.3 (10.4–27.3) |
| HR for OS  (95% CI) *p* value* | 0.420  (0.226–0.781) 0.0049 | | 0.468  (0.264–0.830) 0.0080 | | 0.884 (0.439–1.782) 0.7305 | |
| **ORR**, *n* (%)  (95% CI)  *p* value^†^ | 26 (66.7) (49.8–80.9) | 21 (53.8) (37.2–69.9) | 28 (68.3) (51.9–81.9) | 12 (29.3) (16.1–45.5) | 27 (69.2) (52.4–83.0) | 30 (76.9) (60.7–88.9) |
|  | 0.3548 | | 0.0008 | | 0.6103 | |
| **Difference of ORR**, (%) (95% CI)^‡^  *p* value | 12.82 (-10.590–35.228) 0.3548 | | 39.02 (16.724–58.408) 0.0008 | | -7.69 (-30.415–15.632) 0.6103 | |
| **CR rate as best response**, *n* (%)  (95% CI) *p* value^†^ | 16 (41.0) (25.6–57.9) | 9 (23.1) (11.1–39.3) | 15 (36.6) (22.1–53.1) | 5 (12.2) (4.1–26.2) | 14 (35.9) (21.2–52.8) | 19 (48.7) (32.4–65.2) |
|  | 0.1447 | | 0.0193 | | 0.6103 | |
| **Difference of CR rate**, (%)  (95% CI)^‡^  *p* value | 17.95 (-5.492–39.984) 0.1447 | | 24.39 (1.611–45.353) 0.0193 | | -12.82 (-35.228–10.591) 0.3594 | |
| **Median PFS,** mo (95% CI) | 7.0 (3.6–28.0) | 4.6 (3.0–6.7) | 6.3 (4.3–28.0) | 3.8 (2.6–5.7) | 6.3 (4.3–22.5) | 3.9 (3.1–12.7) |
| HR  (95% CI)  *p* value* | 0.505 (0.271–0.941) 0.0282 | | 0.476 (0.275–0.824) 0.0068 | | 0.661 (0.353–1.238) 0.1940 | |
| **Median DoR**, mo  (95% CI) | 26.1 (4.4–34.8) | 6.1 (2.1–11.3) | 17.7 (3.7–NR) | 12.4 (1.7–19.3) | 17.7 (3.7–34.8) | 5.9 (3.2–NR) |

*Calculated using Log-rank test. ^†^Calculated using Fisher’s exact test. ^‡^Calculated using Chan–Zhang method. Abbreviations: *CAR-T*, CD19 chimeric antigen receptor therapy T-cell; *CI*, confidence interval; *CR*, complete response; *DoR*, duration of response; *HR*, hazard ratio; *MAS*, matched analysis set; *mo*, months; *NR*, not reached; *ORR*, overall response rate; *OS*, overall survival; *PFS*, progression-free survival; *pola-BR*, polatuzumab vedotin + bendamustine + rituximab; *R2*, rituximab + lenalidomide.
